# Secretome and extracellular vesicle signatures in bone marrow-derived mesenchymal stromal cells after expansion in standard and next-generation media

**DOI:** 10.20517/evcna.2024.99

**Published:** 2025-04-29

**Authors:** Giulio Grieco, Simona Piccolo, Enrico Ragni, Laura de Girolamo

**Affiliations:** Laboratorio di Biotecnologie Applicate all’Ortopedia, IRCCS Ospedale Galeazzi - Sant’Ambrogio, Milano I-20157, Italy.; ^#^Authors contributed equally.

**Keywords:** Mesenchymal stromal cells, secretome, extracellular vesicles, miRNA, orthopedics, osteoarthritis

## Abstract

**Aim:** Mesenchymal stem cells (MSCs) are a promising therapeutic strategy for osteoarthritis (OA), largely due to their regenerative potential, which is attributed in part to their secretome. The secretome includes soluble factors and extracellular vesicles (EVs). Given that MSCs are sensitive to various culture conditions, this study aims to investigate the effects of different media supplemented with either fetal bovine serum (FBS) (F), platelet lysate (P), or serum/xeno-free (S/X) on the composition and therapeutic potential of the secretome from bone marrow-derived MSCs (BMSCs).

**Methods:** BMSCs were cultured in F, P, or S/X media, with secretomes collected after starvation. The secretomes were analyzed for soluble factors, EVs, and miRNAs. Inflammatory responses were assessed in an *in vitro* OA model using inflamed chondrocytes and gene expression was evaluated by qRT-PCR.

**Results:** The secretomes from all conditions exhibited a similar molecular fingerprint. Proteomic analysis identified 98 common proteins encompassing growth factors and inflammatory mediators. EVs showed similar size and phenotype, with a slight difference in CD44 expression in EVs derived from P-expanded MSCs. Despite the high overall similarity, miRNA profiling identified 13 key players, with subtle differences in the miRNA composition of EVs from FBS-expanded BMSCs. All secretomes exhibited anti-inflammatory effects, with the FBS-expanded secretome showing the most pronounced therapeutic potential.

**Conclusion:** The secretomes derived from different culture conditions share key molecular components. EVs may contribute to variations in therapeutic outcomes through their cargo. Optimizing MSC expansion conditions is crucial for enhancing the therapeutic potential of MSC-derived secretomes in OA treatment. Further research is needed to clarify the specific role of factors, miRNAs, and EVs in modulating OA pathology.

## INTRODUCTION

In modern orthopedics, the regenerative medicine approach encompasses a wide range of orthobiological options^[1]^, including but not limited to blood-derived products, such as platelet-rich plasma (PRP), and cell-based therapies, such as microfragmented adipose tissue (MFAT), stromal vascular fraction (SVF), or bone marrow aspirate concentrate (BMAC). Current data suggest that orthobiologics are primarily “symptom-modifying”, including the reduction in pain and inflammation, but there is emerging evidence, particularly in preclinical research^[2-5]^, indicating that they can also lead to tissue regeneration (“structure-modifying”)^[6]^. Combining current knowledge with expert opinion, the European Society of Sports Traumatology, Knee Surgery and Arthroscopy (ESSKA) has recently released two consensus documents endorsing both cell-based^[7]^ and blood-derived^[2]^ products as valid options for the conservative treatment of knee osteoarthritis (OA).

The cell-based approach relies on the presence of mesenchymal stromal cells (MSCs) that release an array of bioactive molecules, both free and conveyed within extracellular vesicles (EVs). Under this paradigm, Arnold Caplan suggested changing the name of MSCs to Medicinal Signaling Cells^[8,9]^. To enhance and strengthen their therapeutic impact, the concept of clinically expanded MSCs gained interest in several fields^[10]^, particularly in orthopedics, with a focus on knee OA^[11]^. Of note, nearly 200 MSCs-based clinical trials are currently registered for joint diseases (https://clinicaltrials.gov/, search terms: Condition: Joint Diseases, Other terms: Mesenchymal Stem/Stromal Cells, October 2024), highlighting the increasing interest in this biological approach. Both adipose^[12]^- and bone marrow^[13]^-derived products, whether prepared at the point of care (SVF, MFAT, or BMAC) or culture-expanded [ASCs and bone marrow-derived mesenchymal stromal cells (BMSCs)], have been shown to release soluble proteins and EVs-conveyed miRNAs with anti-inflammatory and cartilage protective properties, collectively defined as “secretome”. Accordingly, MSCs-EVs (NCT06466850) or the whole secretome (NCT05579665 and NCT04314661) are now studied in clinical trials (search terms: Condition: Joint Diseases, Other terms: Mesenchymal Stem/Stromal Cells Extracellular Vesicles/Secretome, October 2024) for OA as cell-free alternatives.

At present, the most standardized protocols for MSC expansions in research related to secretome rely on the use of fetal bovine serum (FBS) as a supplement in culture media. However, this practice raises several concerns. First, the complex composition of FBS includes unknown factors (endotoxins, mycoplasma, viruses, or prion proteins), which may have potential adverse effects that are not adequately standardized and evaluated. Secondly, ethical reasons^[14]^ surrounding the use of animal-derived products are significant. Additionally, if FBS is not completely removed from MSC cultures^[15]^, it may provoke immunologic reactions. While FBS remains widely used in research, its presence in experimental protocols may also lead to inconsistencies, potentially skewing results and leading to false conclusions. Therefore, next-generation media and formulations are actively being studied to eliminate xenogenic contaminants, even in research settings, to ensure more reliable and standardized outcomes when it comes to clinical use. Among these options, human platelet lysate, which is rich in growth factors, cytokines, and plasma, has been extensively used to replace FBS in protocols for the expansion of MSCs under good manufacturing practice (GMP). However, to increase reproducibility - that remains an issue with platelet lysate even if prepared in multi-donors batches - and to reduce the risk of transmission of human diseases^[16]^, an alternative is the use of serum-free and xeno-free chemically defined media (S/X). These media abolish problems such as undefined compositions, variation among plasma units or batches, and the risks of pathogen transmission. Likewise, for these innovative formulations, GMP manufacture and protocols are now readily available^[17]^.

A concern related to using different media and supplements is their effect on MSCs properties. It was reported that in MSCs, platelet lysate and S/X media help maintain a spindle-shaped cell morphology, the expression of typical surface markers, and the capacity of multipotent differentiation and immunomodulation^[18]^. Nevertheless, more detailed analyses showed that in BMSCs, despite meeting the minimal criteria for surface marker expression as defined by the International Society for Cell and Gene Therapy (ISCT)^[19]^, biological differences emerged^[20]^ in both cells and secreted factors during expansion^[21]^ in multiple centers. This lack of knowledge is even more relevant for features and potential of secretomes collected after expansion in various media and supplements, especially in light of the modulations reported after priming strategies, such as inflammation induced by different cytokines^[22]^.

The aim of this work is to assess differences at the molecular level in the secreted factors and EV-conveyed miRNAs fingerprint of secretomes released by BMSCs expanded in either FBS, platelet lysate, or S/X media, and to test their anti-inflammatory properties in an *in vitro* inflammatory OA-model of chondrocytes. Therefore, this study will provide insights into the molecular differences in secreted factors and EV-conveyed miRNAs in BMSC secretomes and will highlight how these variations can influence anti-inflammatory properties in OA treatment, offering valuable translational potential for therapeutic applications in regenerative medicine and the EV field.

## METHODS

### Ethics statement

The study was performed under Institutional Review Board approval (San Raffaele Hospital Ethics Committee approval on 16 December 2020, registered under number 214/int/2020) and Informed Consent administration to patients, following the 1964 Helsinki Declaration and its later amendments or comparable ethical standards.

### BMSCs isolation, culture and secretome preparation

Total bone marrow was collected from 8 donors (2 females and 6 males, mean age: 53 ± 25). Fifty thousand nucleated cells/cm^2^ were seeded and cultivated at 37 °C, 5% CO_2_, and 95% humidity. BMSCs were identified as adherent cells forming colonies and expanded using TrypLE express (ThermoFisher, Waltham, MA, United States) to detach cells when 90% confluence was reached. Three conditions were set:

(i) Condition (F): DMEM/F12 (ThermoFisher) + 10% FBS (GE Healthcare, Piscataway, NJ, USA), 1% L-glutamine plus penicillin-streptomycin (Life Technologies, Carlsbad, CA, USA). (ii) Condition (P): as in i) with 5% human platelet lysate in place of FBS. (iii) Condition (S/X): StemPro™ MSC SFM XenoFree (serum/xeno-free, cGMP compliant) (ThermoFisher), 1% PSG. Before seeding, S/X flasks were coated with CELLstart™ Substrate (serum/xeno-free, cGMP compliant) (ThermoFisher) as per the manufacturer’s instruction.

At passage 2 and 90% confluence, media were removed and three washes with PBS performed to remove residual contaminants. BMSCs were detached and analyzed with a NucleoCounter NC-3000 (Chemometec, Allerod, Denmark) to determine cell number, viability, and size in suspension. BMSCs were suspended in serum-free DMEM/F12 + PSG to reach a 1 × 10^6^ cells/mL concentration for all previously described conditions, and incubated at 37 °C, 5% CO_2_, and 95% humidity. After 48 h, the secretome was collected, centrifuged at 376 × g (1×), 1,000 × g (1×), 2,000 × g (1×), and 4,000 × g (2×) at 4 °C to remove debris. Eventually, each secretome was filtered with a 0.22 μm device, aliquoted and stored at -80 °C until use, when pools for each condition were prepared prior to molecular and functional analyses.

### Flow cytometry

For cells, an aliquot of BMSCs from each of the 8 donors under all three conditions was stained with the following antibodies according to the manufacturer’s instruction for 30 min at 4 °C in the dark: anti-CD90-FITC (code 130-114-859) and CD45-PC7 (130-112-170) (Miltenyi Biotec, Bergisch Gladbach, NRW, Germany); CD271-PE (345105), CD73-APC (344005), and CD146-APCH7 (361027) (BioLegend, San Diego, CA, USA); and CD44-PerCp (44PP2) (Immunostep, Salamanca, Spain). After one wash in FACS buffer, a minimum of 30,000 events were acquired with a CytoFLEX flow cytometer (Beckman Coulter, Fullerton, CA, USA) and processed with CytExpert v2.3 software (Beckman Coulter).

For EVs, three pooled secretomes from the 8 donors were divided into aliquots, diluted in PBS and left unstained, stained with 10 µM carboxyfluorescein succinimidyl ester (CFSE) for 1 h at 37 °C in the dark, or 10 µM CFSE followed by 30 min at 4 °C with the following antibodies, each used separately: anti-CD9-APC (312107), CD63-APC (353007), CD73-APC (344005), CD81-APC (349509), and CD90-APC (321113) (BioLegend). Samples were further 1-fold diluted and at least 10,000 events were acquired with a CytoFLEX flow cytometer (Beckman Coulter) after calibration with FITC-fluorescent nanobeads (100, 160, 200, 300, 240, 500, and 900 nm; Biocytex, Marseille, France) used as an internal control for efficient detection in the nanometric range. Each marker was tested in technical triplicate.

### ELISA assay

The concentration of 200 soluble inflammatory and growth factors, chemokines, receptors, and cytokines was tested in the pooled secretomes from the 8 donors with the Quantibody® Human Cytokine Array 4000 Kit (https://www.raybiotech.com/quantibody-human-cytokine-array-4000/), according to the manufacturer’s instructions (RayBiotech, Norcross, GA, USA). Each factor was tested in quadruplicate. When readings were outside the standard curve values, appropriate dilutions were made. Only factors detected above a single assay threshold in all samples were considered. To obtain the absolute amount of each factor, raw pg/mL concentrations were multiplied per the total volume of secretome, and eventually divided per million cells to obtain a pg/10^6^ BMSCs ratio.

### Nanoparticle tracking analysis

Nanosight NS-300 system (NanoSight Ltd., Amesbury, UK) was used to analyze pooled secretomes from 8 donors (1:3 diluted in PBS). Each analysis was performed in five replicates. Nanoparticle tracking analysis (NTA) software v3.4 provided both concentration measurements and high-resolution particle size distribution profiles.

### miRNA analysis

Identical amounts of pooled secretomes of the 8 donors were 9-fold diluted in PBS for a total volume of 10 mL and ultra-centrifuged at 100,000 × g for 9 h at 4 °C in an Optima L-90K Ultracentrifuge (Beckman Coulter, Brea, CA, USA) equipped with a Type 70.1 Ti Fixed-Angle Titanium Rotor (Beckman Coulter). RNA extraction, cDNA synthesis and qRT-PCR reactions were performed as previously described^[23]^. The global mean method^[24]^ allowed normalization between samples. To monitor whole procedure reproducibility between samples and to assign a quantity to identified miRNAs, ath-miR-159 spike-in was used, comparing its normalized C_RT_ values, corresponding to an input of 30 pg before RNA extraction, with those obtained with each detected miRNA. Analysis for reproducibility was performed in sixteen replicates. Values of each miRNA are reported either as pg per 10^6^ BMSCs or % of single weight with respect to the total weight of the dataset [(pg of a specific miRNA / total pg of all detected miRNAs)^*^100].

### Protein-Protein interaction network generation

The online tool STRING^[25]^ (http://www.string-db.org) (database v12.0) was used to generate interactome maps of ELISA-identified proteins. The following settings were used: (i) organism, Homo sapiens; (ii) meaning of network edges, evidence; (iii) active interaction sources, experiments and databases; (iv) minimum required interaction scores, medium confidence (0.400).

### Clustering analysis

Principal component analysis (PCA) and hierarchical clustering were obtained with the ClustVis^[26]^ package (https://biit.cs.ut.ee/clustvis/). For proteins and miRNA, data were ln(x) transformed. For gene expression, fold change *vs.* CTRL values were used. No row centering was applied. No row scaling was applied. For PCA, singular-value decomposition (SVD) with imputation was used to calculate principal components. For heatmaps, both rows and columns were clustered using correlation distance and average linkage. Other parameters were: correlation for clustering distance for rows/columns and average for clustering methods for rows/columns. For tree ordering for columns and rows, the tightest cluster first option was applied.

### EV-miRNA target identification

The miRNet web tool (https://www.mirnet.ca/miRNet/home.xhtml, database v2.0) was employed to retrieve the EV-miRNA targets, on the following settings: (i) organism, Homo sapiens; (ii) ID type, miRBase ID; (iii) tissue, not specified; (iv) targets, genes (miRTarBase v9.0). Options *Include PPI (gene only)* and *Include tf2gene* were not flagged. The parameters set for the *Function Explorer* section were: (i) query, All miRNAs; (ii) Algorithm, Hypergeometric test; (iii) Database, miRNA Disease and miRNA Function. Only target genes validated by robust experimental evidence (reporter assay, Western blot, and qPCR) were selected. To facilitate visual exploration and functional interpretation of the miRNA-target interaction network, we employed the miRNet Linear Bipartite/Tripartite layout.

### Functional experiment on chondrocytes

Immortalized chondrocytes (InSCREENex GmbH, Braunschweig, Germany. The cell line is discontinued)^[27]^ at p10 were seeded in complete DMEM/F12. For the experiment, the following conditions were set: complete DMEM/F12, complete DMEM/F12 + 1 ng/ml IL1B (Interleukin 1β, to simulate the inflammatory process observed in OA) (Sino Biological, Eschborn, Germany), complete DMEM/F12 + pooled secretomes from the 8 donors (F, P, and S/X), complete DMEM/F12 + 1 ng/ml IL1B + pooled secretomes from the 8 donors (F, P, and S/X). The final concentration of the secretomes was 50% and the final concentration of FBS was 10% in each well. After 48h, viability was determined by automated fluorescence cytometry relying on fluorescent dyes, acridine orange, and DAPI, to identify live and dead cells (NucleoCounter® NC-3000™; Chemometech, Allerod, Denmark). Cell number was measured by nucleic acid quantification (CyQUANT^®^; ThermoFisher) following the manufacturer’s instructions. Eventually, for RNA retrieval, cells were harvested with QIAzol (Qiagen, Hilden, Germany) and stored at -20 °C for molecular analyses. RNA was extracted with miRNeasy Micro Kit (Qiagen) and the same amount of RNA was retrotranscribed in cDNA using the iScriptTM cDNA synthesis Kit (BioRad, Hercules, CA, USA). The amplifications were conducted using the iTaq Universal SYBR Green Supermix (BioRad) in a CFX Opus Real-Time PCR Systems (BioRad), according to the manufacturer’s instruction. The analyzed genes were: *IL1B* (Interleukin 1β), *IL6* (Interleukin 6), *IL8* (Interleukin 8), *CTSS* (Cathepsin S), *CCL2* (C-C Motif Chemokine Ligand 2), *CCL5* (C-C Motif Chemokine Ligand 5), *BCL2* (B-cell CLL/lymphoma 2), and *BAX* (BCL2 associated X). Primer sequences will be provided upon request. The relative expression levels of target genes were assessed with the fold change (2^−ΔΔCt^) method, using *TBP* (TATA-Binding Protein) as the housekeeping gene to standardize targets’ mRNA concentrations.

### Statistical analysis

Statistical analyses were conducted using Prism 8 software (GraphPad Software, La Jolla, CA, USA). The ROUT test was used to identify outliers (Q = 0.1%). Normal distribution was calculated with a Shapiro-Wilk normality test and an alpha level of 0.01. For whole population analyses and biological replicates, with normally distributed data, a repeated measures paired ANOVA analysis was performed. With non-normally distributed data, paired Friedman test was performed. For technical replicates, with normally distributed data, an ordinary one-way unpaired ANOVA analysis was performed. With non-normally distributed data, unpaired Kruskal-Wallis test was performed. A significance threshold of p ≤ 0.05 was applied and values below this threshold were considered statistically significant. For correlation analyses, if the normality test was passed, a Pearson correlation coefficient was calculated; otherwise, a nonparametric Spearman correlation coefficient was computed, both two-tailed with a 95% confidence interval. The R-squared calculation was used to generate the square of Pearson’s product moment correlation coefficient through data points.

## RESULTS

### BMSC characterization and immunophenotype

**Figure 1:**
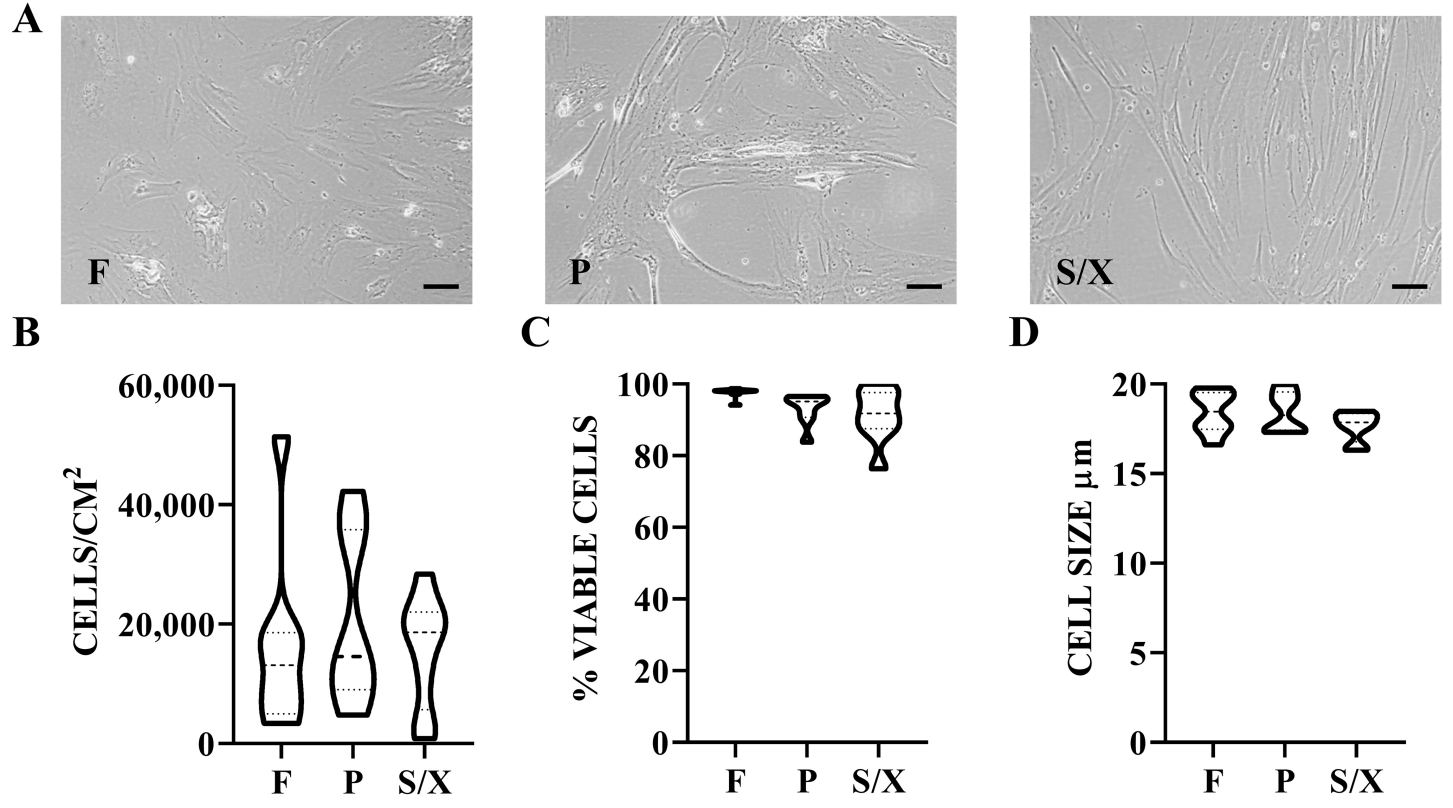
BMSCs phenotype in analyzed media. (A) BMSCs showed fibroblast-like morphology with a more elongated shape in P and S/X conditions. Scale bar = 10 µm; (B) Violin plots of BMSCs density, *N* = 8 donors. Median and quartiles are shown as dashed and dotted lines, respectively; (C) Violin plots of BMSCs viability, *N* = 8 donors; (D) Violin plots showing the size distribution of BMSCs in suspension after trypsin detachment and before secretome production, *N* = 8 donors. Median and quartiles are shown as dashed and dotted lines, respectively. BMSCs: Bone marrow-derived mesenchymal stromal cells; F: fetal bovine serum; P: platelet lysate; S/X: serum/xeno free.

**Figure 2:**
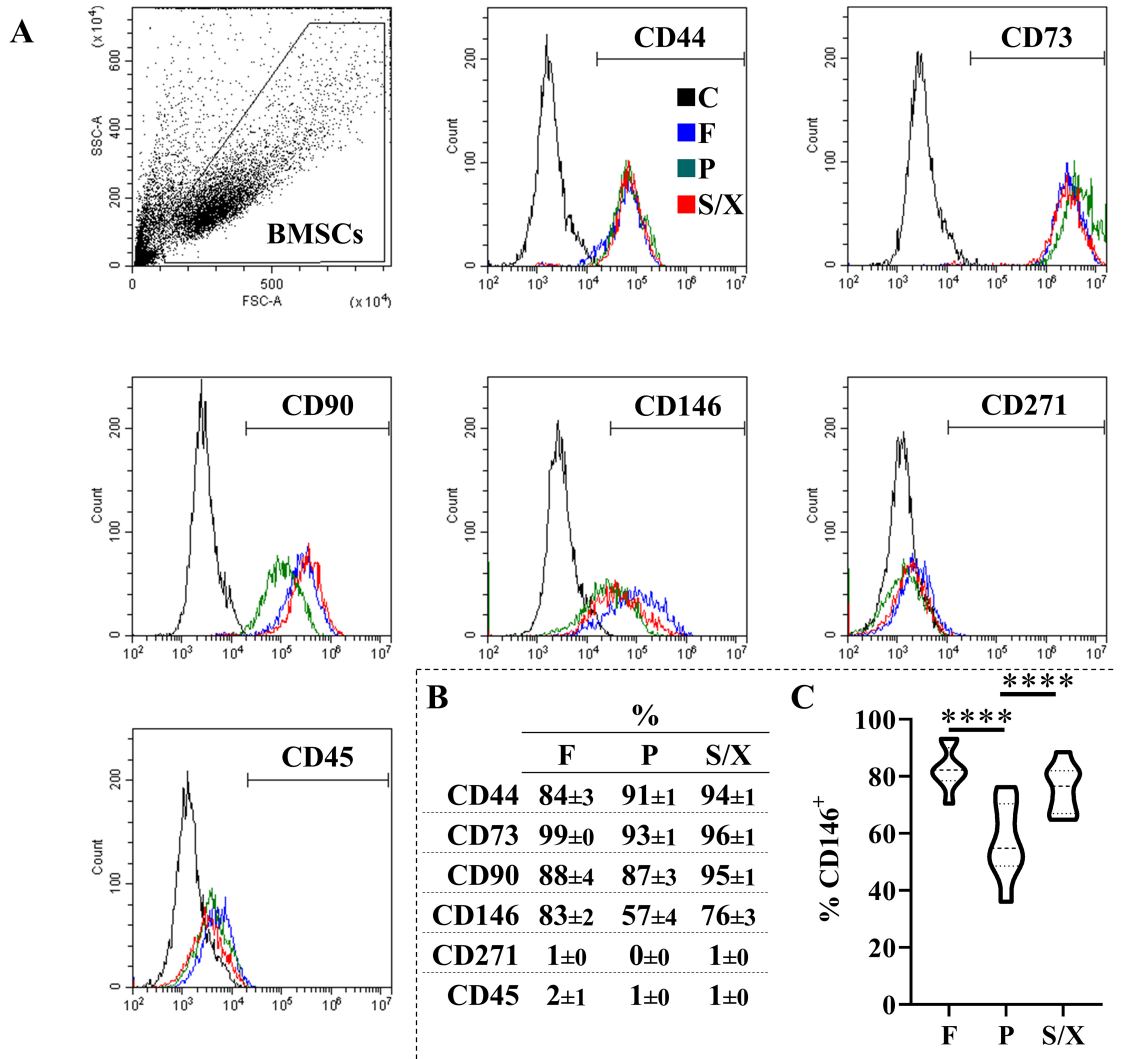
Immunophenotype of BMSCs in analyzed media. (A and B) BMSCs in the different media were positive for CD44, CD73, CD90, and CD146, while negative for CD271 and CD45. In the histograms, only BMSCs in the F condition are shown for clarity. In the Abs staining plots, BMSCs were gated using the FSC *vs.* SSC. Data shown are from a representative donor; (C) Violin plot of CD146 positivity, *N* = 8 donors. Median and quartiles are shown as dashed and dotted lines, respectively. BMSCs: Bone marrow-derived mesenchymal stromal cells; F: fetal bovine serum; P: platelet lysate; S/X: serum/xeno free; FSC: forward scatter; SSC: side scatter. ^****^P-value ≤ 0.0001.

BMSCs showed typical fibroblast-like morphology, with a more elongated phenotype in P and S/X [Figure 1A]. Cell density was 16.2 cells/cm^2^ × 10^3^ cells/cm^2^ ± 5.1 in F, 20.8 ± 4.8 in P, and 15.7 ± 3.2 in S/X (mean ± SEM, *N* = 8) [Figure 1B]. Viability was 97.6% ± 0.5% for cells cultured in F, 93.1 ± 1.4 for those cultured in P, and 91.2 ± 2.5 for S/X [Figure 1C]. Cell size after detachment was 18.4 µM ± 0.4 in F and P conditions, and 17.7 ± 0.3 in S/X [Figure 1D]. No significant difference emerged for all these parameters among different culture conditions. Regarding surface markers, BMSCs were always consistently (> 80%) positive for MSC markers CD44, CD73, and CD90 and negative for CD45 and CD271 regardless of the culture media [Figure 2A and B]. CD146 was the most divergent marker, showing significantly lower expression in P (57% ± 4%) compared to F (83% ± 2%) and S/X (76% ± 3%) [Figure 2C].

### BMSC secreted factors

**Figure 3:**
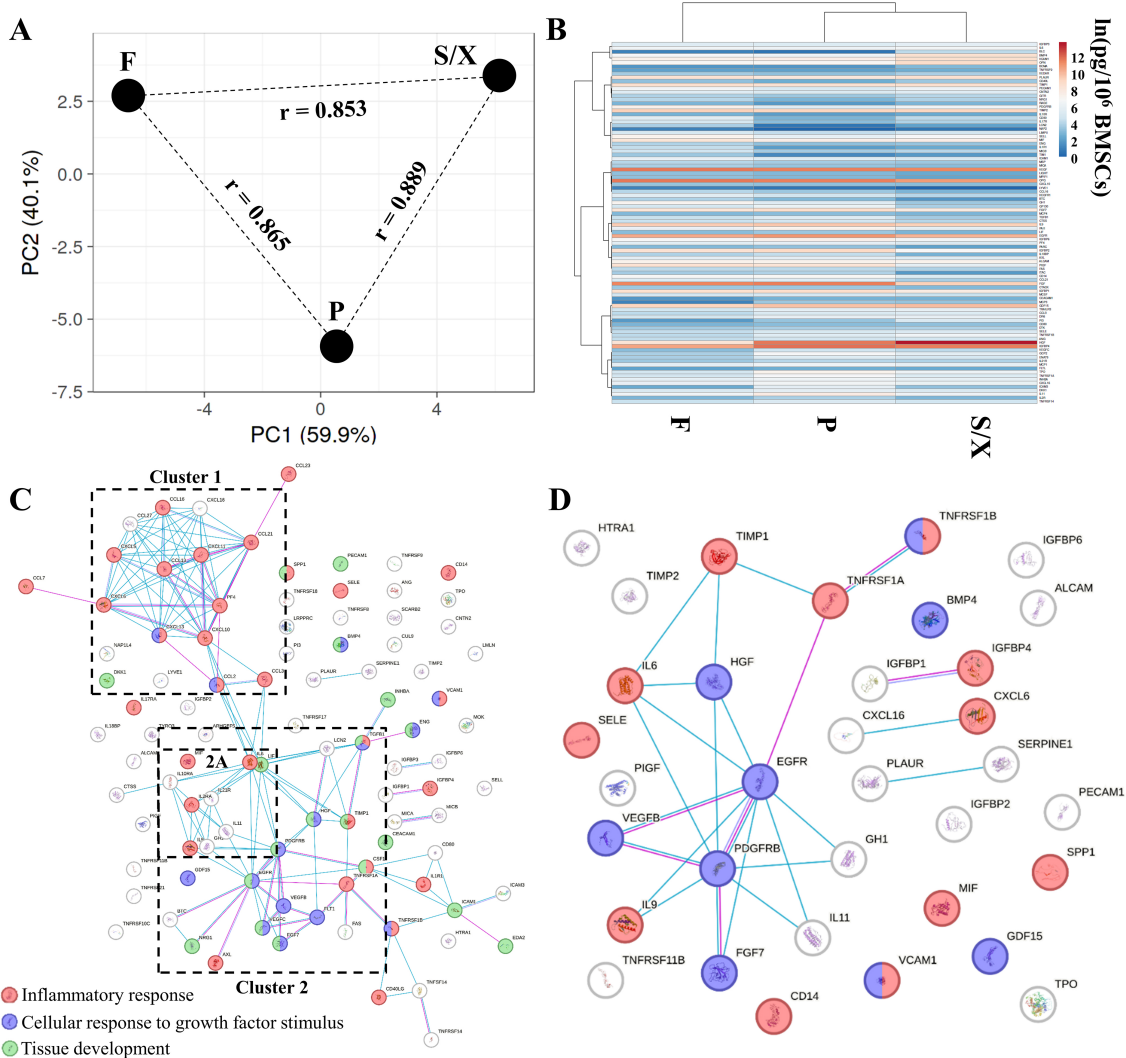
BMSC-secreted factors after expansion in the analyzed media. (A) Principal component analysis of the ln(x) transformed pg/10^6^ BMSCs values for the detected factors. The X- and Y-axes represent principal components 1 and 2 that explain 59.9% and 40.1% of the total variance, respectively. F stands for FBS, P for hPL, and S/X for serum/xeno free. “r” denotes the Spearman correlation coefficient; (B) Heatmap of hierarchical clustering analysis of the ln(x) transformed pg/10^6^ BMSCs values of detected factors, with sample clustering tree shown at the top. The color scale indicates absolute expression levels: red shades = high expression levels and blue shades = low expression levels. F, P, and S/X represent the same media conditions as in panel A; (C) Functional association network of the identified secreted factors, generated using the online tool STRING. Blue connections are for proteins with known interactions based on curated databases; violet connections for proteins with experimentally determined interactions. Empty nodes, proteins with unknown 3D structure; filled nodes, proteins with known or predicted 3D structure; (D) Functional association network of secreted factors in the first quartile of abundance, also generated using the online tool STRING. Color coding and graphic elements are consistent with (C). BMSCs: Bone marrow-derived mesenchymal stromal cells; F: fetal bovine serum; P: platelet lysate; S/X: serum/xeno free.

A total of 98 soluble proteins were shared by all the secretomes obtained after BMSCs’ expansion in the three media [Supplementary Table 1]. Spearman R correlation values were high and similar among the three conditions (0.87 for F *vs.* P, 0.85 for F *vs.* S/X, and 0.89 for P *vs.* S/X). The pattern was corroborated by the PCA plot [Figure 3A], where samples appeared equidistant, with greater similarity confirmed for P and S/X, which were positioned under the same node (Figure 3B, magnified in Supplementary Figure 1). The most abundant (≥ 100,000 pg/10^6^ BMSCs) factors were VEGF (154,080), OPG (152,943), and FGF (113,264) for F; IGFBP4 (167,261), HGF (151,329), FGF (127,456), VEGF (126,217), and OPG (122,678) for P; HGF (1,122,590), IGFBP4 (130,876), and VEGF (111,268) for S/X. Of note, further supporting the high similarity between conditions, 19 factors were shared in the first quartile of abundance across all three secretomes [Table 1], representing the core set of proteins released by BMSCs, regardless of the expansion media used before secretome production. The most diverging factors found in the first quartile in one condition compared to the others were CXCL16, GCP2, PAI1, TPO, TNFR1 for P, TNFR2 for S/X, and GH1 for F. In contrast, several growth factors, insulin-like binding proteins (IGFBP1/2/4/6), and tissue inhibitors of metalloproteinases (TIMP1/2) were shared. Accordingly, overall, when considering each condition as a whole, no statistically significant difference emerged, reinforcing the correlation analysis results for the entire dataset.

**Table 1:** Soluble factors released by BMSCs after expansion in the three media under study, in the first quartile of abundance

**pg/10^6^ BMSCs**			
	F	P	S/X
ALCAM	**1,756**	**1,753**	**1,670**
BMP4	**915**	**693**	**6,948**
CD14	**1,034**	**1,081**	213
CXCL16	438	**784**	502
EGFR	**44,699**	**73,317**	**32,263**
FGF	**113,264**	**127,456**	**13,605**
FGF7	**4,882**	**8,043**	**2,837**
GCP2	49	**809**	292
GDF15	**11,565**	**21,261**	**23,174**
GH1	**1,479**	263	34
HGF	**5,383**	**151,329**	**1,122,590**
IGFBP1	**7,408**	**8,042**	**2,996**
IGFBP2	**5,933**	**11,195**	**1,018**
IGFBP4	**48,777**	**167,261**	**130,876**
IGFBP6	**1,035**	**1,864**	**719**
IL11	**636**	**2,738**	561
IL6	**642**	593	**710**
IL9	**15,748**	**18,331**	**13,687**
MIF	**4,395**	**2,165**	**2,360**
OPG	**152,943**	**122,678**	**62,970**
OPN	**1,114**	**1,200**	**7,705**
PAI1	458	**1,735**	299
PDGFRB	**1,951**	262	**616**
PECAM1	**797**	192	**605**
PIGF	**5,773**	**5,543**	319
PLAUR	**6,095**	**4,121**	**7,081**
SELE	**1,725**	391	**886**
TIMP1	**6,926**	**6,363**	**7,110**
TIMP2	**13,113**	**11,968**	**12,438**
TNFR1	124	**825**	209
TNFR2	67	239	**637**
TPO	127	**1,810**	590
VCAM1	**1,905**	**2,195**	**6,622**
VEGF	**154,080**	**126,217**	**111,268**

In bold, factors in the first quartile of abundance for each condition. BMSCs: Bone marrow-derived mesenchymal stromal cells; F: fetal bovine serum; P: platelet lysate; S/X: serum/xeno free.

A protein association network analysis was performed to assign an overall function to the detected factors [Figure 3C]. Two main clusters emerged. A tighter one (Cluster 1, magnified in Supplementary Figure 2) mainly consisted of factors involved in Inflammatory response (GO:0006954), including several C-C motif chemokine ligands (CCL2/3/7/13/16/21/23) and C-X-C motif chemokine ligands (CXCL5/6/10/11/13). The second cluster (Cluster 2, magnified in Supplementary Figure 3) was looser, with fewer inter-factor connections, and was driven by Cellular response to growth factor stimulus (GO:0071363) and Tissue development (GO:0009888). Notably, several factors were involved in both biological processes (BP) (EGFR, FGF7, HGF, PDGFRB, and VEGFC). A small sub-cluster (Cluster 2A) was defined by inflammation-related cytokines and their receptors (IL6/9/11 and IL2RA/10RA/21R). Finally, a network analysis of the most abundant proteins in the first quartile of expression confirmed the presence of a core network defined by the cellular response to growth factor stimulus (EGFR, FGF7, HGF, PDGFRB, and VEGFB) as the preponderant message regardless of the expansion media used before secretome collection [Figure 3D].

### EVs characterization

**Figure 4:**
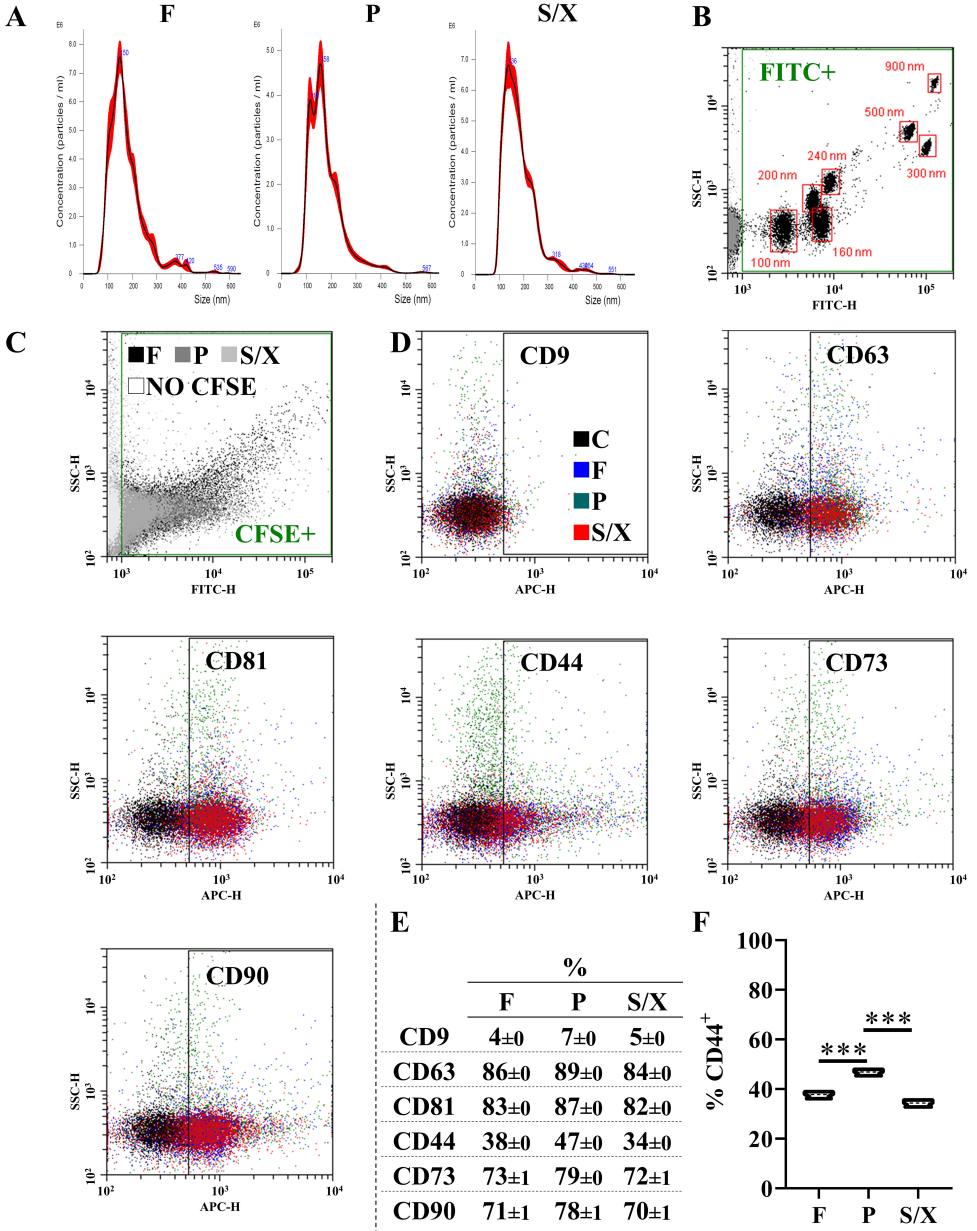
EV phenotype in pooled secretomes. (A) Nanoparticle tracking analysis of pooled secretomes obtained after cultivating BMSCs in F, P, and S/X media; (B) Flow cytometer analysis of FITC-fluorescent nanobeads (100 - 160 - 200 - 240 - 300 - 500 - 900 nm) to ensure correct visualization of particles within the study range (< 100 nm up to 1 µm); (C) Flow cytometer detection of CFSE-labeled EVs compared to unstained EVs. Only unstained EVs in F secretomes are shown for readability. A representative plot is shown; (D and E) EVs in the different media were positive for EV markers CD63 and CD81, and MSC lineage markers CD73 and CD90. CD9, another EV marker, was almost absent, while CD44 (an MSC-lineage marker) was dim. Only unstained CFSE-EVs from the F condition are visualized for readability. A representative plot per Ab is shown; (F) Violin plot of CD44 positivity, *N* = 3 pools (each from 8 donors). Median and quartiles are shown as dashed and dotted lines, respectively. BMSCs: Bone marrow-derived mesenchymal stromal cells; F: fetal bovine serum; P: platelet lysate; S/X: serum/xeno free; EVs: extracellular vesicles; CFSE: carboxyfluorescein succinimidyl ester; MSC: mesenchymal stromal cells. ^***^P-value ≤ 0.001.

EVs in the pooled secretomes showed similar physical features, with comparable sizes (Mean: 177 nm ± 1 for F, 180 ± 1 for P, 182 ± 1 for S/X; Mode: 154 ± 1 for F, 138 ± 4 for P, 146 ± 3 for S/X (mean ± SEM, *N* = 5 NTA analysis) [Figure 4A]. A flow cytometer was calibrated with FITC-fluorescent beads (from 100 to 900 nm) to ensure EV detection within the nanometric range [Figure 4B]. As shown in the plot, fluorescent CFSE-labeled EVs were detected with sizes comparable to those observed by NTA, mainly ranging from < 100 nm to 300 nm with few particles up to 500 nm [Figure 4C]. EVs exhibited a similar immunophenotype, with < 10% positive for CD9, > 80% for EVs markers CD63/81, and > 70% for the lineage markers CD73/90 [Figure 4D and E]. CD44, another MSC lineage marker, was dim (30%-40%), with a higher amount in P secretomes [Figure 4F].

### BMSC-EVs embedded miRNAs

**Figure 5:**
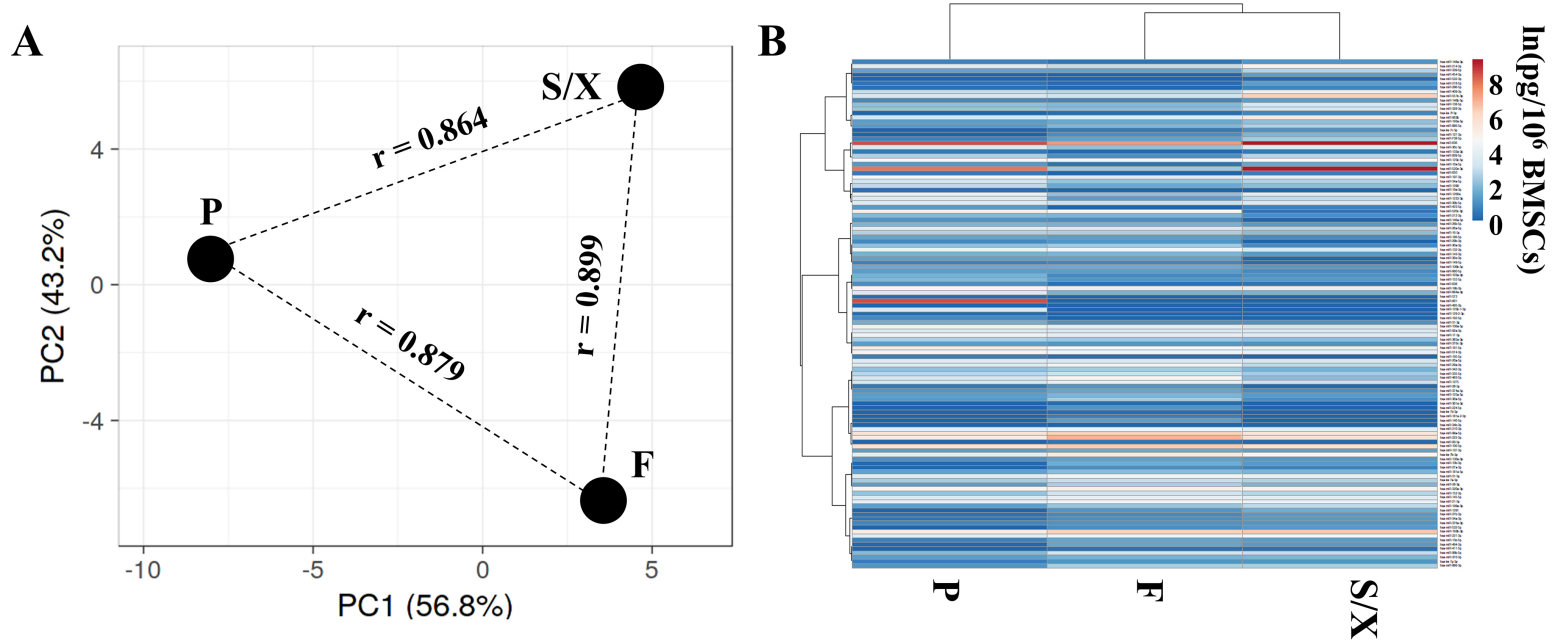
EV-miRNAs from BMSCs after expansion in the analyzed media. (A) Principal component analysis of the ln(x) transformed pg/10^6^ BMSCs values for detected EV-miRNAs. The X- and Y-axes represent principal components 1 and 2 that explain 56.8% and 43.2% of the total variance, respectively. “r” represents the Spearman correlation coefficient; (B) Heatmap of hierarchical clustering analysis of the ln(x) transformed pg/10^6^ BMSCs values of detected EV-miRNAs, with sample clustering tree shown at the top. The color scale reflects the absolute expression levels: red shades: high expression levels; blue shades: low expression levels. F, P, and S/X represent the same media conditions as in (A). BMSCs: Bone marrow-derived mesenchymal stromal cells; F: fetal bovine serum; P: platelet lysate; S/X: serum/xeno free; EVs: extracellular vesicles.

A total of 165 miRNAs were commonly found in EVs obtained from BMSC secretomes following expansion in all three media [Supplementary Table 2]. Spearman R correlation coefficients were high and comparable across the three conditions: 0.88 for F *vs.* P, 0.90 for F *vs.* S/X, and 0.86 for P *vs.* S/X. The pattern was corroborated by the PCA plot [Figure 5A], where samples were equidistant, and greater similarity was confirmed between F and S/X that clustered under the same node (Figure 5B, magnified in Supplementary Figure 4). The most abundant miRNAs (≥ 1,000 pg/10^6^ BMSCs) were: hsa-miR-636 (1,613) and hsa-miR-222-3p (1,015) for F; hsa-miR-601 (4,818), hsa-miR-636 (4,759), and hsa-miR-520e-3p (2,729) for P; hsa-miR-520e-3p (10,982) and hsa-miR-636 (10,528) for S/X. Notably, further supporting the great similarity between conditions, 5 miRNAs were shared among all three EV types in the group representing > 1% of the total genetic weight [Table 2], suggesting a stable core of EV-miRNAs released by BMSCs regardless of the expansion media. Two miRNAs were specific to both F and P (hsa-miR-520c-3p, hsa-miR-191-5p) and one was shared by P and S/X (hsa-miR-520e-3p). The most divergent miRNAs, each contributing > 1% of genetic weight in only one condition, were hsa-miR-601 in P and hsa-miR-551b-3p in S/X. In F, several miRNAs slightly exceeded 1% while less abundant (< 1%) in P and S/X, although their amount (in pg per million BMSCs) remained in a comparable range (dozens to hundreds of pg). Overall, when considering each condition as a whole, no statistically significant differences were observed, reinforcing the results of the correlation analysis across the dataset.

**Table 2:** EV-miRNAs released by BMSCs after expansion in the three media under study, with > 1% genetic weight

	**F**		**P**		**S/X**	
	pg/10^6^ BMSCs	%	pg/10^6^ BMSCs	%	pg/10^6^ BMSCs	%
hsa-miR-636	**1,614.0**	**24.0**	**4,759.7**	**30.5**	**10,528.2**	**40.7**
hsa-miR-222-3p	**1,015.8**	**15.1**	**218.4**	**1.4**	**324.5**	**1.3**
hsa-miR-100-5p	**616.3**	**9.2**	**320.4**	**2.1**	**352.4**	**1.4**
hsa-miR-99a-5p	**612.4**	**9.1**	**296.7**	**1.9**	**280.9**	**1.1**
hsa-miR-193b-3p	**560.0**	**8.3**	**202.2**	**1.3**	**682.6**	**2.6**
hsa-let-7b-5p	**234.0**	**3.5**	42.1	0.3	118.4	0.5
hsa-miR-320a-3p	**168.1**	**2.5**	121.6	0.8	142.5	0.6
hsa-miR-483-5p	**121.5**	**1.8**	27.1	0.2	7.9	0.03
hsa-miR-520c-3p	**98.6**	**1.5**	**175.4**	**1.1**	1.6	0.0
hsa-miR-191-5p	**91.3**	**1.4**	**201.5**	**1.3**	58.0	0.2
hsa-miR-132-3p	**84.8**	**1.3**	64.6	0.4	26.2	0.1
hsa-miR-125b-5p	**81.7**	**1.2**	105.8	0.7	110.7	0.4
hsa-miR-31-5p	**80.0**	**1.2**	41.6	0.3	63.8	0.2
hsa-miR-21-5p	**77.0**	**1.1**	51.8	0.3	63.8	0.2
hsa-miR-29a-3p	**76.3**	**1.1**	35.2	0.2	16.6	0.1
hsa-miR-574-3p	**75.9**	**1.1**	126.3	0.8	56.1	0.2
hsa-miR-221-3p	**72.4**	**1.1**	45.1	0.3	80.6	0.3
hsa-miR-210-3p	**67.7**	**1.0**	46.5	0.3	44.1	0.2
hsa-miR-551b-3p	22.8	0.3	31.0	0.2	**613.9**	**2.4**
hsa-miR-520e-3p	10.6	0.2	**2,729.9**	**17.5**	**10,982.9**	**42.5**
hsa-miR-601	0.3	0.004	**4,818.6**	**30.9**	0.3	0.001

In bold, miRNAs with ≥ 1% for each condition. BMSCs: Bone marrow-derived mesenchymal stromal cells; F: fetal bovine serum; P: platelet lysate; S/X: serum/xeno free; EVs: extracellular vesicles.

To assign a role to identified EV-miRNAs, the miRNet tool was used on the whole dataset. Intriguingly, several miRNAs were found to be connected with the Disease term “Osteoarthritis” [Table 3]. Narrowing the search for BP known to be altered in OA, we found chondrocyte differentiation (GO: 0002062) and development (GO: 0002063), and, most importantly, immune (GO: 0006955) and inflammatory (GO: 0006954) response. Consistently, the last two were characterized by the greatest number of miRNAs, 30 and 33, respectively, while chondrocyte-related BP terms were defined by 9 and 12, respectively. F condition had the highest amount and % for the majority of identified categories. Nevertheless, in the view of assigning a preferential impact for one condition over the others, it has to be noted that miRNAs with both high and low amounts are included. Additionally, the different miRNAs may have positive or negative effects on the whole process, making the understanding of the whole contribution unpredictable. Thus, we focused our attention on those miRNAs falling in the > 1% weight group [Table 2].

**Table 3:** EV-miRNAs associated with OA-related terms and biological processes identified by miRNet

**Osteoarthritis**	**Inflammatory response**	**Immune response**	**Chondrocyte development**	**Chondrogenic differentiation**
	**GO: 0006954**	**GO: 0006955**	**GO: 0002063**	**GO: 0002062**
hsa-mir-21-5p	hsa-let-7d-5p	hsa-let-7g-5p	hsa-mir-34a-3p	hsa-mir-29a-3p
hsa-mir-26b-5p	hsa-let-7f-5p	hsa-mir-16-5p	hsa-mir-34a-5p	hsa-mir-30a-3p
hsa-mir-27a-3p	hsa-let-7g-5p	hsa-mir-27b-3p	hsa-mir-92a-3p	hsa-mir-30a-5p
hsa-mir-27a-5p	hsa-mir-20b-5p	hsa-mir-27b-5p	hsa-mir-145-3p	hsa-mir-140-3p
hsa-mir-27b-3p	hsa-mir-210-3p	hsa-mir-29b-3p	hsa-mir-145-5p	hsa-mir-140-5p
hsa-mir-27b-5p	hsa-mir-27b-3p	hsa-mir-30a-3p	hsa-mir-146a-5p	hsa-mir-181a-2-3p
hsa-mir-30b-5p	hsa-mir-27b-5p	hsa-mir-30a-5p	hsa-mir-181a-2-3p	hsa-mir-181a-3p
hsa-mir-335-5p	hsa-mir-34a-3p	hsa-mir-92a-3p	hsa-mir-181a-3p	hsa-mir-181a-5p
hsa-mir-34a-3p	hsa-mir-34a-5p	hsa-mir-100-5p	hsa-mir-181a-5p	hsa-mir-495-3p
hsa-mir-34a-5p	hsa-mir-708-5p	hsa-mir-103a-3p	hsa-mir-181c-5p	
hsa-mir-125b-1-3p	hsa-mir-744-5p	hsa-mir-125b-1-3p	hsa-mir-210-3p	
hsa-mir-125b-5p	hsa-mir-99b-3p	hsa-mir-125b-5p	hsa-mir-222-3p	
hsa-mir-127-3p	hsa-mir-99b-5p	hsa-mir-126-3p		
hsa-mir-130a-3p	hsa-mir-100-5p	hsa-mir-127-3p		
hsa-mir-139-5p	hsa-mir-125a-5p	hsa-mir-132-3p		
hsa-mir-140-3p	hsa-mir-125b-1-3p	hsa-mir-146a-5p		
hsa-mir-140-5p	hsa-mir-125b-5p	hsa-mir-146b-5p		
hsa-mir-145-3p	hsa-mir-126-3p	hsa-mir-155-5p		
hsa-mir-145-5p	hsa-mir-132-3p	hsa-mir-181a-2-3p		
hsa-mir-146a-5p	hsa-mir-133a-3p	hsa-mir-181a-3p		
hsa-mir-146b-5p	hsa-mir-134-5p	hsa-mir-181a-5p		
hsa-mir-148a-3p	hsa-mir-138-5p	hsa-mir-181c-5p		
hsa-mir-181a-2-3p	hsa-mir-146b-5p	hsa-mir-186-5p		
hsa-mir-181a-3p	hsa-mir-155-5p	hsa-mir-192-5p		
hsa-mir-181a-5p	hsa-mir-181a-2-3p	hsa-mir-19b-3p		
hsa-mir-181c-5p	hsa-mir-181a-3p	hsa-mir-203a-3p		
hsa-mir-199a-3p	hsa-mir-181a-5p	hsa-mir-210-3p		
hsa-mir-204-5p	hsa-mir-192-5p	hsa-mir-370-3p		
hsa-mir-365a-3p	hsa-mir-193b-3p	hsa-mir-532-3p		
hsa-mir-370-3p	hsa-mir-203a-3p	hsa-mir-532-5p		
	hsa-mir-221-3p			
	hsa-mir-222-3p			
	hsa-mir-335-5p			
pg/10^6^ BMSCs				
334	2,601	1,019	1,194	115
352	1,122	763	363	54
346	1,741	674	467	30
% genetic weight				
5.0	38.8	15.2	17.8	1.7
2.3	7.2	4.9	2.3	0.4
1.3	6.7	2.6	1.8	0.1

BMSCs: Bone marrow-derived mesenchymal stromal cells; EVs: extracellular vesicles; OA: osteoarthritis.

**Figure 6:**
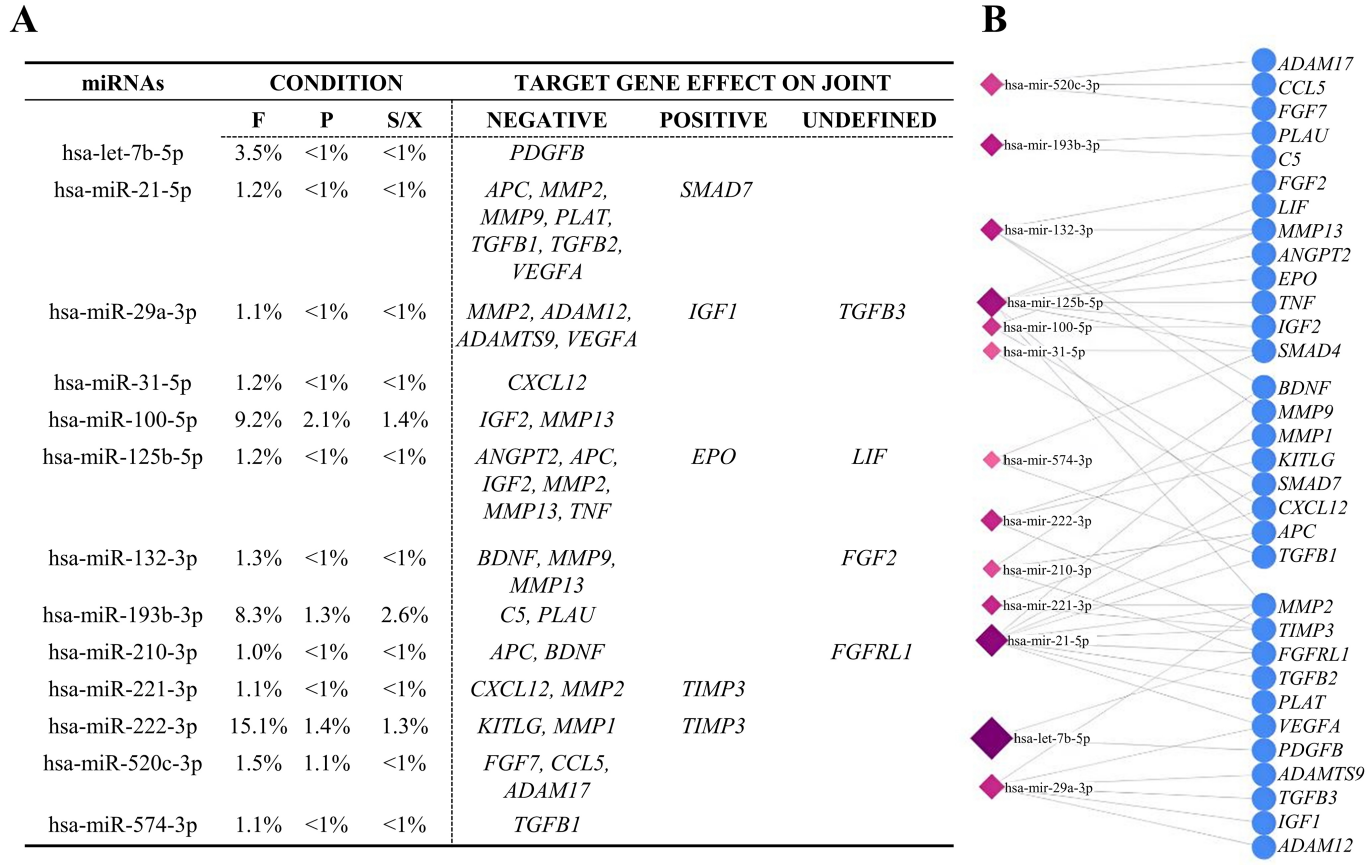
OA-related target genes of the most abundant miRNAs. (A) miRNAs that represent ≥ 1% of the total in at least one condition target genes known to be modulated in OA tissues. % indicates each miRNA’s relative abundance under each condition; (B) The identified genes may be targeted by more than one miRNA. OA: Osteoarthritis; F: fetal bovine serum; P: platelet lysate; S/X: serum/xeno free.

Of the 21 analyzed miRNAs, 17 were reported to have experimentally validated targets [Supplementary Table 3]. Narrowing the analysis for factors reported as dysregulated in OA specimens, such as cytokines/chemokines, growth factors, or matrix remodeling^[28]^, 29 players regulated by 13 miRNAs were identified [Figure 6A]. Notably, 20 of these candidates have a negative effect on the joint, 5 are protective and 4 have dual roles depending on the specific isoforms or disease stage. The F condition was again characterized by players with higher amounts, driven by hsa-miR-222-3p (15.1%, 1,016 pg / 10^6^ BMSCs), hsa-miR-100-5p (9.2%, 616), and hsa-miR-193b-3p (8.3%, 560). It is worth noting that, similar to miRNAs targeting multiple genes, individual genes may also be targeted by more than one miRNA [Figure 6B].

### Effect of secretomes on an in vitro inflammatory OA model of chondrocytes

**Figure 7:**
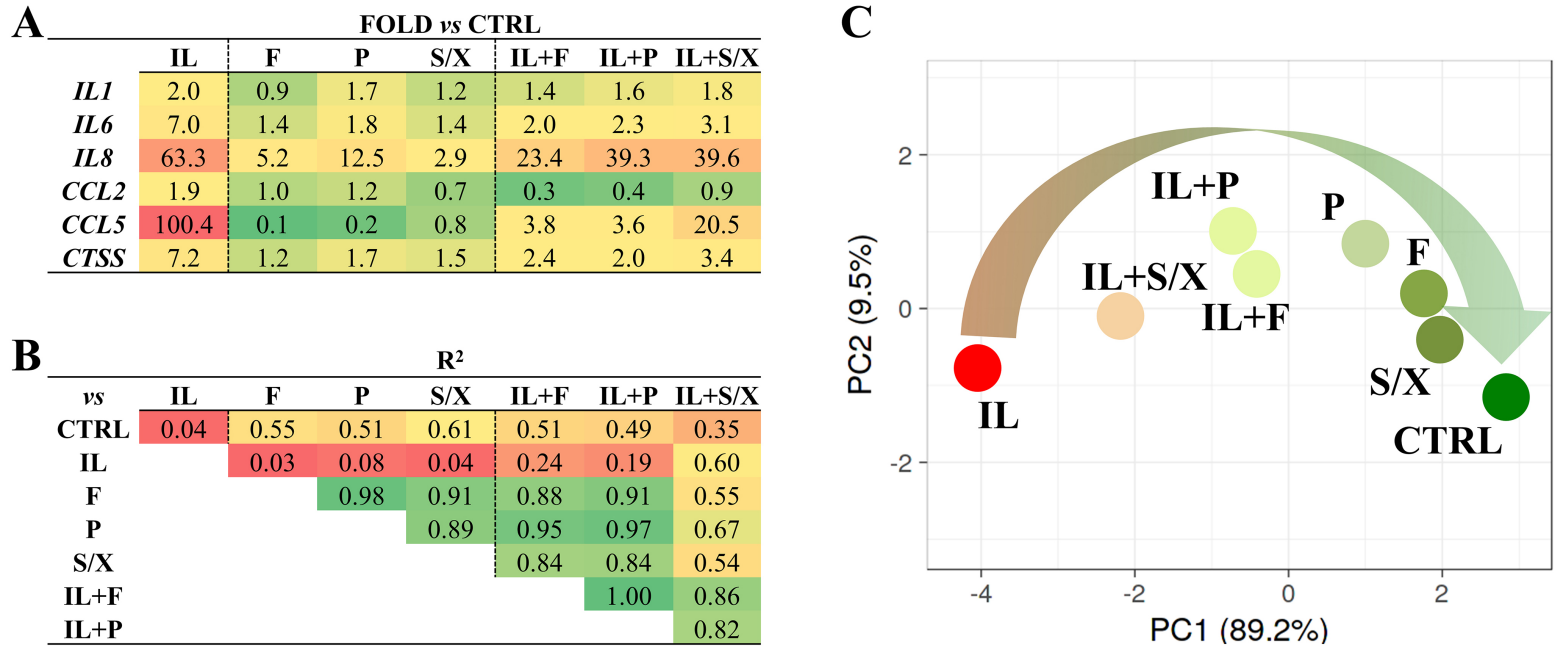
Effects of the secretome on inflamed chondrocytes. (A) Gene expression modulation in chondrocytes either untreated or treated with IL, and subsequently untreated or treated with F, P, and S/X secretomes (pooled from 8 donors each). Values are reported as fold relative to untreated (CTRL) cells. The color code ranges from green (downregulation) to red (upregulation) relative to CTRL. (B) R-squared correlation values for the gene expression ratios for the different conditions in chondrocytes, either untreated or treated with IL and secretomes (F, P, S/X). The color scale ranges from red (low correlation) to green (high correlation). (C) Principal component analysis of gene expression modulation as shown in (A). The X- and Y-axes represent principal components 1 and 2 that explain 83.2% and 9.5% of the total variance, respectively. IL: Interleukin 1β-treated cells. F: fetal bovine serum; P: platelet lysate; S/X: serum/xeno free.

The effects of F, P, and S/X secretomes were tested on human chondrocytes with and without Interleukin 1β to mimic the OA phenotype. First, no differences were observed in cell proliferation rate, viability, or the *BCL2*/*BAX* ratio, whose reduction is linked to increased cell susceptibility to apoptosis [Supplementary Table 4]. Next, a panel of inflammation-triggered genes was analyzed [Figure 7A], encompassing inflammatory cytokines (*IL1/6/8*), chemokines (*CCL2/5*), and a protease (*CTSS*) involved in the inflammation-driven degradation of cartilage extra cellular matrix (ECM), all characteristic of arthritic conditions. In control cells (without IL1β), treatment with the secretomes did not alter gene expression, except for *IL8*, which was upregulated by all three secretomes, and *CCL5*, which was downregulated by F and P. In inflamed cells, all secretomes were able to downregulate the expression of the tested genes, including *IL8. CCL2* expression remained steady in the presence of IL1β, although it was reduced compared to CTRL. Among the treatments, secretome F showed the most pronounced effects, followed by P, while S/X had the weakest impact, as indicated by the coefficient of determination R^2^ [Figure 7B]. These results were further confirmed by PCA analysis: the IL + S/X group was the closest to IL chondrocytes, whereas the IL + F and IL + S groups were the nearest to not-inflamed cells [Figure 7C].

## DISCUSSION

The main findings of the study reveal that the molecular fingerprints of soluble factors and EV-conveyed miRNAs, altogether called secretome, released by BMSCs expanded in different culture conditions (F, P, and S/X) exhibit similar overall characteristics. All three secretomes demonstrated anti-inflammatory potential in an *in vitro* model of OA chondrocytes, although some divergences were observed, with the FBS condition showing a slight advantage.

It is well established that the composition and impact of the MSC-derived secretome are significantly influenced by culture conditions^[29]^, including the type of medium, cell density, viability, and size^[29]^. Recent studies underscore the need to optimize and standardize culture protocols to maximize the regenerative potential of MSC-derived secretomes for clinical use^[30]^.

BMSCs expanded in FBS, platelet lysate, or S/X media maintained their typical fibroblast-like morphology and homogeneity in all above-mentioned parameters. Regardless of the culture media, BMSCs expressed specific markers such as CD44, CD73, and CD90, in line with established literature that defines the minimal criteria for identifying MSCs^[19]^. The main immunophenotypic difference was a lower expression of CD146 in condition P in contrast to F and S/X, although violin plots in Figure 2C showed partial overlap. Of note, CD146^+^ BMSCs were reported to have more pronounced secretory and immunomodulatory properties^[31]^, further explored in the study. This difference may be attributed to the media composition, as platelet lysate contains high levels of bFGF^[32]^, which can suppress CD146 expression^[33]^. Future studies are needed to further investigate how the media and supplement composition modulate the MSC phenotype.

A robust profile of 98 proteins among chemokines, cytokines, and growth factors was identified across all conditions, finding a high degree of overlap also for this parameter. The observed consistency is particularly evident in scoring the subset of the most abundant, and perhaps effective, proteins in the first quartile of abundance, indicating a shared fundamental set regardless of the expansion media used before secretome production. In this group laid several growth factors, together with insulin-like growth factor-binding protein (IGFBPs) and tissue inhibitors of metalloproteinases (TIMPs), all of which have been reported to play roles in the OA environment. Numerous growth factors are known to promote cell proliferation, cartilage regeneration, and, in particular, exert anti-apoptotic effects on chondroblasts^[34]^. Accordingly, preparations enriched in growth factors are currently utilized in OA treatment due to their significant anti-inflammatory and analgesic properties. In this frame, IGFBPs bind to, shuttle, and possibly protect insulin-like growth factors (IGFs) from degradation in the articular space. IGFs, structurally related to pro-insulin, promote chondrocyte proliferation, enhance matrix production, and inhibit apoptosis^[35]^. TIMPs help maintain joint homeostasis^[36]^, but in OA, matrix metalloproteinases (MMPs) often outweigh TIMPs, making supplementation a potential therapy. Other factors such as BMP4^[37]^, OPN^[38]^, and OPG^[39,40]^ (via the RANKL/OPG ratio) further suggest how BMSC secretome may influence OA-altered processes. Correspondingly, protein network analysis of the scored factors identified clusters linked to inflammation, growth factor response, and tissue development - key pathways known to be dysregulated in OA. In particular, inflammatory mediators were notably abundant and are especially relevant given the evolving understanding of OA. Once considered a cartilage-driven disease, OA is now recognized as a multifaceted condition involving inflammatory mediators released by cartilage, bone, and synovium^[41]^. Consequently, biologic-based OA therapies aim not only to modulate matrix homeostasis but also to manage inflammation and alleviate pain - core therapeutic objectives, highlighting the importance of evaluating their anti-inflammatory potential, rather than focusing solely on matrix regulation as classically done. It is worth noting that the few molecules found to differ between conditions were insufficient to support a clear preference for one product over the others in terms of therapeutic efficacy.

To shed further light on possible differences between F, P, and S/X secretomes, EVs were analyzed. In fact, there is mounting evidence that the therapeutic potential of MSCs lies in the ability of EVs and their cargoes to control immune cell homing, phenotype, and function^[42]^. Moreover, in OA, MSC-EVs can regulate diseased tissue homeostasis such as chondrocyte function and metabolic dysregulation, thereby mitigating disease progression^[43]^. NTA revealed comparable EV size and physical features across all conditions. EV immunophenotyping confirmed a high degree of similarity in specific markers CD63, CD81, CD73, and CD90. The only notable difference, despite a similar overall expression (30%-50% of analyzed particles), was a higher CD44 level in P EVs. Since MSC-EVs bind to hyaluronic acid via CD44^[44]^, even a small variation could influence EV bioavailability in joints, where hyaluronan is a key synovial fluid component. Further research in this direction is needed to assess the impact on the therapeutic features. Subsequently, a comprehensive analysis of 165 detected EV-miRNAs showed high similarity across conditions, as evidenced by correlation values, PCA, and heat mapping. This suggests a shared regulatory network, as observed for soluble factors. Several miRNAs were associated with OA-related processes in the miRNet database, including chondrocyte differentiation and immune/inflammatory responses. These latter pathways involved the highest number of miRNAs, with F EVs exhibiting the greatest number and abundance. However, a definitive role for EV-embedded miRNAs remains unclear, as they may exert both protective and pathological effects. A focused analysis identified miRNAs that account for 1% of the total genetic weight in at least one condition and have validated OA-related targets. Thirteen miRNAs were found to regulate 29 genes - 20 with pathological roles and only five protective. Thus, the inhibitory action of these miRNAs may mitigate OA progression by reducing inflammation, inhibiting aberrant signaling, and supporting tissue integrity, ultimately contributing to disease attenuation. Consistent with this, *in vivo* studies have demonstrated positive outcomes when MSC-EVs are administered to degenerated cartilage tissue in the hopes of encouraging regeneration^[45]^. Notably, the miRNAs analyzed in this study are significantly enriched in the F secretome compared to the other conditions, where their expression levels are near the defined threshold. Their preferential abundance in the F secretome may indicate a unique regulatory profile specific to this condition, potentially driven by high levels of hsa-miR-222-3p, hsa-miR-100-5p, and hsa-miR-193b-3p. According to miRNet, all three miRNAs are associated with Inflammatory/Immune response BP, with hsa-miR-222-3p also linked to Chondrocyte development. Specifically, hsa-miR-222-3p serves as a marker for M2b macrophages and positively regulates innate immune responses^[46]^, while inhibiting metalloproteinases^[47]^. hsa-miR-100-5p reduces synovial cell proliferation and inflammation^[48]^, and hsa-miR-193b-3p is a marker for M2a anti-inflammatory macrophages^[49]^. Notably, hsa-miR-222-3p and hsa-miR-193b-3p are downregulated in OA chondrocytes^[47]^ and cartilage^[50]^, respectively, whereas hsa-miR-100-5p, shuttled by MSC-EVs, helps protect articular cartilage from OA damage^[51]^. These findings suggest that variations in EV cargo composition may influence BMSC secretome’s capacity to promote healing in pathological chondrocytes, with a particular focus on inflammation as evidenced by soluble factors. This notion reinforces the need for tests to evaluate the anti-inflammatory potential of biological products.

In this context, a functional assay was conducted to evaluate the therapeutic efficacy of the secretomes using an *in vitro* model of inflamed chondrocytes that mimics the OA phenotype. All BMSC-derived secretomes effectively downregulated genes associated with inflammation (*IL1, IL6, IL8, CCL2, CCL5, CTSS*). This effect was particularly pronounced with the F secretome, which demonstrated the greatest efficacy in reducing the expression of inflammation-triggered genes. PCA clustering consistently reflected this trend, revealing that the IL + F condition most closely resembled the non-inflamed state, whereas the IL + P and IL + S/X conditions exhibited less pronounced effects. Interestingly, when the secretomes in the absence of inflammation were tested, a weak increase in *IL8* gene expression was observed across all three conditions, alongside a reduction in *CCL5* expression in F and P. It can be hypothesized that the secretomes alone may activate specific inflammatory pathways, although this mechanism appears to be suppressed or even reversed in the presence of IL-1β. These findings indicate a potential interplay between the bioactive components of the secretomes, as those identified within soluble factors and EV-shuttled miRNAs, and the inflammatory microenvironment, highlighting the context-dependent nature of their effects on cellular signaling pathways. Further studies and an enlarged panel of genes are needed to confirm these observations.

This study has limitations. First, secretomes from different BMSC donors were pooled before analyses. While this approach likely reduced sample variability and improved representativeness by combining secretomes released by eight distinct BMSC donors, it may have masked individual donor-specific effects. Further experiments to validate the results using individual samples are planned as part of a dedicated project. Second, the proteomic analysis of the soluble fraction of the secretomes was confined to a targeted panel specifically associated with inflammatory pathways. Consequently, other potentially relevant factors outside this scope may have been overlooked and warrant further investigation. Similarly, the analysis of miRNAs was limited to a panel of well-characterized candidates. While this facilitates the functional interpretation of each detected miRNA, it also excludes less well-understood miRNAs that could provide additional insights. Lastly, the study employed a basic 2D *in vitro* model primarily designed to assess chondrocyte responses to inflammation, offering only a partial representation of the OA phenotype in patient tissues. While 2D systems serve as a first-line approach for evaluating the efficacy of new drugs or compounds, they lack the niche environment necessary to assess matrix homeostasis and the modulation of anabolic and catabolic factors - features that are better replicated in 3D models. Furthermore, the use of immortalized chondrocytes derived from a healthy donor - to reduce inter-donor variability - rather than primary cells from OA patients, and their stimulation with an inflammatory agent, may further limit the physiological relevance of the findings since immortalized cell lines are typically less susceptible to modulation of proliferation or cell death/apoptosis. Future studies employing 3D models with primary OA chondrocytes are needed to better characterize the effects of secretomes, yielding insights that more accurately reflect patient physiology at both the matrix and inflammatory response levels.

In conclusion, this study offers valuable insights into the therapeutic potential of BMSCs for OA treatment, with a focus on the secretome they release under different culture conditions. The findings demonstrate that BMSC secretomes, irrespective of the culture medium, exhibit significant anti-inflammatory potential in OA chondrocyte models, with some variations favoring FBS. The study underscores the importance of optimizing culture conditions to enhance the regenerative potential of MSC-derived secretomes for clinical applications. Notable findings include the identification of proteins associated with OA, which could promote cartilage regeneration, reduce apoptosis, and balance joint homeostasis. Additionally, EVs containing miRNAs play a crucial role in modulating immune responses and influencing tissue repair. These results emphasize the translational value of MSC secretomes in OA therapy, reinforcing the need for further research to optimize and assess their anti-inflammatory properties.

## DECLARATIONS

### Availability of data and materials

The datasets generated and/or analyzed during the current study are available in the Open Science Framework repository, https://osf.io/ps4aw/?view_only=1cfa5202b1e4414f85a4490fb9f3e32e

### Copyright

© The Author(s) 2025.

## References

[1] Mavrogenis AF, Karampikas V, Zikopoulos A (2023). Orthobiologics: a review. Int Orthop.

[3] Perucca Orfei C, Boffa A, Sourugeon Y (2023). Cell-based therapies have disease-modifying effects on osteoarthritis in animal models. A systematic review by the ESSKA orthobiologic initiative. Part 1: adipose tissue-derived cell-based injectable therapies. Knee Surg Sports Traumatol Arthrosc.

[4] Boffa A, Perucca Orfei C, Sourugeon Y (2023). Cell-based therapies have disease-modifying effects on osteoarthritis in animal models. A systematic review by the ESSKA orthobiologic initiative. Part 2: bone marrow-derived cell-based injectable therapies. Knee Surg Sports Traumatol Arthrosc.

[5] Boffa A, Salerno M, Merli G (2021). Platelet-rich plasma injections induce disease-modifying effects in the treatment of osteoarthritis in animal models. Knee Surg Sports Traumatol Arthrosc.

[6] Rodeo SA (2023). Orthobiologics: current status in 2023 and future outlook. J Am Acad Orthop Surg.

[7] https://cdn.ymaws.com/www.esska.org/resource/resmgr/docs/consensus_projects/2024_orbit_complete_report.pdf.

[8] Caplan AI, Correa D (2011). The MSC: an injury drugstore. Cell Stem Cell.

[9] Caplan AI (2017). Mesenchymal stem cells: time to change the name!. Stem Cells Transl Med.

[10] Miclau K, Hambright WS, Huard J, Stoddart MJ, Bahney CS (2023). Cellular expansion of MSCs: shifting the regenerative potential. Aging Cell.

[11] Harrison-Brown M, Scholes C, Hafsi K (2019). Efficacy and safety of culture-expanded, mesenchymal stem/stromal cells for the treatment of knee osteoarthritis: a systematic review protocol. J Orthop Surg Res.

[12] Ragni E, Perucca Orfei C, De Luca P (2020). Inflammatory priming enhances mesenchymal stromal cell secretome potential as a clinical product for regenerative medicine approaches through secreted factors and EV-miRNAs: the example of joint disease. Stem Cell Res Ther.

[13] Ragni E, Perucca Orfei C, de Girolamo L (2022). Secreted factors and extracellular vesicles account for the immunomodulatory and tissue regenerative properties of bone-marrow-derived mesenchymal stromal cells for osteoarthritis. Cells.

[14] Gstraunthaler G, Lindl T, van der Valk J (2013). A plea to reduce or replace fetal bovine serum in cell culture media. Cytotechnology.

[15] Rowland AL, Burns ME, Levine GJ, Watts AE (2021). Preparation technique affects recipient immune targeting of autologous mesenchymal stem cells. Front Vet Sci.

[16] Iudicone P, Fioravanti D, Bonanno G (2014). Pathogen-free, plasma-poor platelet lysate and expansion of human mesenchymal stem cells. J Transl Med.

[17] Aussel C, Busson E, Vantomme H, Peltzer J, Martinaud C (2022). Quality assessment of a serum and xenofree medium for the expansion of human GMP-grade mesenchymal stromal cells. PeerJ.

[18] Bui HTH, Nguyen LT, Than UTT (2021). Influences of Xeno-free media on mesenchymal stem cell expansion for clinical application. Tissue Eng Regen Med.

[19] Dominici M, Le Blanc K, Mueller I (2006). Minimal criteria for defining multipotent mesenchymal stromal cells. The international society for cellular therapy position statement. Cytotherapy.

[20] Fitzgerald JC, Shaw G, Murphy JM, Barry F (2023). Media matters: culture medium-dependent hypervariable phenotype of mesenchymal stromal cells. Stem Cell Res Ther.

[21] Liu S, de Castro LF, Jin P (2017). Manufacturing differences affect human bone marrow stromal cell characteristics and function: comparison of production methods and products from multiple centers. Sci Rep.

[22] Giannasi C, Della Morte E, Cadelano F (2024). Boosting the therapeutic potential of cell secretome against osteoarthritis: comparison of cytokine-based priming strategies. Biomed Pharmacother.

[23] Ragni E, Colombini A, Viganò M (2021). Cartilage protective and immunomodulatory features of osteoarthritis synovial fluid-treated adipose-derived mesenchymal stem cells secreted factors and extracellular vesicles-embedded miRNAs. Cells.

[24] D’haene B, Mestdagh P, Hellemans J, Vandesompele J (2012). miRNA expression profiling: from reference genes to global mean normalization. Methods Mol Biol..

[25] Szklarczyk D, Kirsch R, Koutrouli M (2023). The STRING database in 2023: protein-protein association networks and functional enrichment analyses for any sequenced genome of interest. Nucleic Acids Res.

[26] Metsalu T, Vilo J (2015). ClustVis: a web tool for visualizing clustering of multivariate data using Principal Component Analysis and heatmap. Nucleic Acids Res.

[27] Lipps C, Klein F, Wahlicht T (2018). Expansion of functional personalized cells with specific transgene combinations. Nat Commun.

[28] Chou CH, Jain V, Gibson J (2020). Synovial cell cross-talk with cartilage plays a major role in the pathogenesis of osteoarthritis. Sci Rep.

[29] Chouaib B, Desoutter A, Cuisinier F, Collart-Dutilleul PY (2025). Dental pulp stem cell conditioned medium enhance osteoblastic differentiation and bone regeneration. Stem Cell Rev Rep.

[30] Su Y, Xu C, Cheng W, Zhao Y, Sui L, Zhao Y (2023). Pretreated mesenchymal stem cells and their secretome: enhanced immunotherapeutic strategies. Int J Mol Sci.

[31] Bowles AC, Kouroupis D, Willman MA, Perucca Orfei C, Agarwal A, Correa D (2020). Signature quality attributes of CD146^+^ mesenchymal stem/stromal cells correlate with high therapeutic and secretory potency. Stem Cells.

[32] Cheng NC, Tu YK, Lee NH, Young TH (2020). Influence of human platelet lysate on extracellular matrix deposition and cellular characteristics in adipose-derived stem cell sheets. Front Cell Dev Biol.

[33] Gharibi B, Hughes FJ (2012). Effects of medium supplements on proliferation, differentiation potential, and in vitro expansion of mesenchymal stem cells. Stem Cells Transl Med.

[34] Civinini R, Nistri L, Martini C, Redl B, Ristori G, Innocenti M (2013). Growth factors in the treatment of early osteoarthritis. Clin Cases Miner Bone Metab.

[35] Wen C, Xu L, Xu X, Wang D, Liang Y, Duan L (2021). Insulin-like growth factor-1 in articular cartilage repair for osteoarthritis treatment. Arthritis Res Ther.

[36] Su S, Grover J, Roughley PJ (1999). Expression of the tissue inhibitor of metalloproteinases (TIMP) gene family in normal and osteoarthritic joints. Rheumatol Int.

[37] Deng ZH, Li YS, Gao X, Lei GH, Huard J (2018). Bone morphogenetic proteins for articular cartilage regeneration. Osteoarthritis Cartilage.

[38] Luo W, Lin Z, Yuan Y, Wu Z, Zhong W, Liu Q (2023). Osteopontin (OPN) alleviates the progression of osteoarthritis by promoting the anabolism of chondrocytes. Genes Dis.

[39] Tat SK, Pelletier JP, Velasco CR, Padrines M, Martel-Pelletier J (2009). New perspective in osteoarthritis: the OPG and RANKL system as a potential therapeutic target?. Keio J Med.

[40] Di Cicco G, Marzano E, Mastrostefano A (2024). The pathogenetic role of RANK/RANKL/OPG signaling in osteoarthritis and related targeted therapies. Biomedicines.

[41] Zhu R, Fang H, Wang J (2024). Inflammation as a therapeutic target for osteoarthritis: a literature review of clinical trials. Clin Rheumatol.

[42] Harrell CR, Jovicic N, Djonov V, Arsenijevic N, Volarevic V (2019). Mesenchymal stem cell-derived exosomes and other extracellular vesicles as new remedies in the therapy of inflammatory diseases. Cells.

[43] You B, Zhou C, Yang Y (2023). MSC-EVs alleviate osteoarthritis by regulating microenvironmental cells in the articular cavity and maintaining cartilage matrix homeostasis. Ageing Res Rev.

[44] Zhou L, Hao Q, Sugita S (2021). Role of CD44 in increasing the potency of mesenchymal stem cell extracellular vesicles by hyaluronic acid in severe pneumonia. Stem Cell Res Ther.

[45] Jones M, Jones E, Kouroupis D (2024). The use of mesenchymal stem/stromal cell-derived extracellular vesicles in the treatment of osteoarthritis: insights from preclinical studies. Bioengineering.

[46] Nejad C, Stunden HJ, Gantier MP (2018). A guide to miRNAs in inflammation and innate immune responses. FEBS J.

[47] Song J, Jin EH, Kim D, Kim KY, Chun CH, Jin EJ (2015). MicroRNA-222 regulates MMP-13 via targeting HDAC-4 during osteoarthritis pathogenesis. BBA Clin.

[48] Liu H, Chen Y, Huang Y (2024). Macrophage-derived mir-100-5p orchestrates synovial proliferation and inflammation in rheumatoid arthritis through mTOR signaling. J Nanobiotechnology.

[49] Graff JW, Dickson AM, Clay G, McCaffrey AP, Wilson ME (2012). Identifying functional microRNAs in macrophages with polarized phenotypes. J Biol Chem.

[50] Meng F, Li Z, Zhang Z (2018). MicroRNA-193b-3p regulates chondrogenesis and chondrocyte metabolism by targeting HDAC3. Theranostics.

[51] Wu J, Kuang L, Chen C (2019). miR-100-5p-abundant exosomes derived from infrapatellar fat pad MSCs protect articular cartilage and ameliorate gait abnormalities via inhibition of mTOR in osteoarthritis. Biomaterials.

