# Supplementary material for: Secretome and extracellular vesicle signatures in bone marrow-derived mesenchymal stromal cells after expansion in standard and next-generation media
Source: Extracell Vesicles Circ Nucl Acids. 2025 Apr 29;6(2):195–215. doi: 10.20517/evcna.2024.99 (PMC12367461; doi:10.20517/evcna.2024.99)
Supplement: Supplementary file 1 [file evcna-6-2-195-SupplementaryMaterials.zip › evcna5099-SupplementaryMaterials/Supplementary Table 1_EVCNA.docx]

**Supplementary Table 1. Soluble factors released by BMSCs after expansion in the three media under study**

|  | **pg/10^6^ BMSCs** | | |
| --- | --- | --- | --- |
|  | **F** | **P** | **S/X** |
| ALCAM | 1756 | 1753 | 1670 |
| ANG | 82 | 120 | 146 |
| AXL | 341 | 327 | 219 |
| BCMA | 6 | 6 | 19 |
| BLC | 3 | 2 | 71 |
| BMP4 | 915 | 693 | 6948 |
| BTC | 51 | 20 | 6 |
| CCL16 | 62 | 33 | 17 |
| CCL21 | 227 | 255 | 43 |
| CCL3 | 72 | 199 | 205 |
| CD14 | 1034 | 1081 | 213 |
| CD30 | 289 | 18 | 68 |
| CD40L | 26 | 13 | 33 |
| CD80 | 18 | 24 | 27 |
| CEACAM1 | 6 | 16 | 15 |
| CNTN2 | 229 | 190 | 235 |
| CTACK | 33 | 35 | 19 |
| CTSS | 87 | 125 | 62 |
| CXCL10 | 31 | 28 | 24 |
| CXCL16 | 438 | 784 | 502 |
| DKK1 | 185 | 317 | 178 |
| DR6 | 131 | 407 | 442 |
| DTK | 38 | 48 | 52 |
| EGFR | 44699 | 73317 | 32263 |
| ENA78 | 44 | 218 | 136 |
| ENG | 56 | 26 | 29 |
| FAS | 68 | 71 | 55 |
| FGF | 113264 | 127456 | 13605 |
| FGF7 | 4882 | 8043 | 2837 |
| FLTL | 9 | 13 | 11 |
| GCP2 | 49 | 809 | 292 |
| GDF15 | 11565 | 21261 | 23174 |
| GH1 | 1479 | 263 | 34 |
| GITR | 53 | 34 | 57 |
| GP130 | 148 | 466 | 46 |
| HGF | 5383 | 151329 | 1122590 |
| ICAM1 | 442 | 234 | 170 |
| ICAM3 | 12 | 328 | 27 |
| IGFBP1 | 7408 | 8042 | 2996 |
| IGFBP2 | 5933 | 11195 | 1018 |
| IGFBP3 | 252 | 209 | 347 |
| IGFBP4 | 48777 | 167261 | 130876 |
| IGFBP6 | 1035 | 1864 | 719 |
| IL10R | 16 | 8 | 11 |
| IL11 | 636 | 2738 | 561 |
| IL17R | 146 | 22 | 56 |
| IL18BP | 54 | 82 | 16 |
| IL1R1 | 84 | 8 | 11 |
| IL21R | 37 | 64 | 54 |
| IL2R | 17 | 47 | 18 |
| IL6 | 642 | 593 | 710 |
| IL9 | 15748 | 18331 | 13687 |
| INHBA | 347 | 541 | 382 |
| ITAC | 40 | 52 | 7 |
| LCN2 | 44 | 2 | 9 |
| LIF | 22 | 37 | 17 |
| LIGHT | 27 | 21 | 11 |
| LIMPII | 154 | 83 | 117 |
| LYVE1 | 3 | 2 | 1 |
| MCP1 | 305 | 529 | 388 |
| MCP3 | 3 | 35 | 31 |
| MCP4 | 20 | 22 | 18 |
| MCSF | 130 | 133 | 99 |
| MICA | 46 | 30 | 23 |
| MICB | 114 | 40 | 57 |
| MIF | 4395 | 2165 | 2360 |
| MPIF1 | 27 | 21 | 10 |
| MSP | 57 | 41 | 34 |
| NAP2 | 3 | 1 | 2 |
| NRG1 | 60 | 18 | 56 |
| OPG | 152943 | 122678 | 62970 |
| OPN | 1114 | 1200 | 7705 |
| PAI1 | 458 | 1735 | 299 |
| PARC | 20 | 25 | 9 |
| PDGFRB | 1951 | 262 | 616 |
| PECAM1 | 797 | 192 | 605 |
| PF4 | 272 | 335 | 179 |
| PI3 | 7 | 31 | 199 |
| PIGF | 5773 | 5543 | 319 |
| PLAUR | 6095 | 4121 | 7081 |
| RAGE | 18 | 7 | 18 |
| SELE | 83 | 152 | 181 |
| SELL | 1725 | 391 | 886 |
| TGFB1 | 91 | 230 | 42 |
| TIM1 | 34 | 4 | 7 |
| TIMP1 | 6926 | 6363 | 7110 |
| TIMP2 | 13113 | 11968 | 12438 |
| TNFRSF14 | 96 | 176 | 101 |
| TNFRSF1A | 124 | 825 | 209 |
| TNFRSF1B | 67 | 239 | 637 |
| TNFRSF9 | 9 | 9 | 14 |
| TPO | 127 | 1810 | 590 |
| TRAILR3 | 108 | 306 | 358 |
| VCAM1 | 1905 | 2195 | 6622 |
| VEGF | 154080 | 126217 | 111268 |
| VEGFC | 24 | 103 | 72 |
| VEGFR1 | 297 | 131 | 59 |
| XEDAR | 40 | 28 | 49 |

F stands for FBS, P for hPL and S/X for serum/xeno-free medium.
