# Supplementary material for: Secretome and extracellular vesicle signatures in bone marrow-derived mesenchymal stromal cells after expansion in standard and next-generation media
Source: Extracell Vesicles Circ Nucl Acids. 2025 Apr 29;6(2):195–215. doi: 10.20517/evcna.2024.99 (PMC12367461; doi:10.20517/evcna.2024.99)
Supplement: Supplementary file 1 [file evcna-6-2-195-SupplementaryMaterials.zip › evcna5099-SupplementaryMaterials/Supplementary Table 2_EVCNA.docx]

**Supplementary Table 2. miRNAs embedded in BMSC-EVs after expansion in the three media under study**

|  | **pg/10^6^ BMSCs** | | |  | **% on the total EV-miRNAs weight** | | | |
| --- | --- | --- | --- | --- | --- | --- | --- | --- |
|  | **F** | **P** | **S/X** |  | **F** | **P** | **S/X** |  |
| hsa-let-7a-5p | 18.41 | 11.94 | 14.05 |  | 0.2743 | 0.0765 | 0.0543 |  |
| hsa-let-7b-5p | 234.00 | 42.07 | 118.35 |  | 3.4861 | 0.2695 | 0.4576 |  |
| hsa-let-7c-5p | 1.72 | 0.30 | 3.05 |  | 0.0256 | 0.0019 | 0.0118 |  |
| hsa-let-7d-5p | 1.26 | 0.11 | 0.83 |  | 0.0188 | 0.0007 | 0.0032 |  |
| hsa-let-7f-5p | 1.21 | 0.62 | 2.17 |  | 0.0181 | 0.0040 | 0.0084 |  |
| hsa-let-7g-5p | 3.21 | 1.87 | 3.22 |  | 0.0478 | 0.0119 | 0.0125 |  |
| hsa-miR-100-5p | 616.26 | 320.38 | 352.38 |  | 9.1807 | 2.0521 | 1.3624 |  |
| hsa-miR-103a-3p | 2.36 | 5.96 | 2.89 |  | 0.0352 | 0.0382 | 0.0112 |  |
| hsa-miR-106a-5p | 48.79 | 70.55 | 27.92 |  | 0.7268 | 0.4519 | 0.1079 |  |
| hsa-miR-106b-5p | 5.14 | 5.08 | 3.50 |  | 0.0766 | 0.0325 | 0.0135 |  |
| hsa-miR-10a-5p | 1.52 | 3.49 | 4.48 |  | 0.0227 | 0.0223 | 0.0173 |  |
| hsa-miR-10b-3p | 5.65 | 1.17 | 3.38 |  | 0.0841 | 0.0075 | 0.0131 |  |
| hsa-miR-10b-5p | 0.09 | 0.02 | 0.07 |  | 0.0013 | 0.0001 | 0.0003 |  |
| hsa-miR-1227-3p | 0.11 | 0.89 | 0.65 |  | 0.0016 | 0.0057 | 0.0025 |  |
| hsa-miR-1233-3p | 3.72 | 31.90 | 17.38 |  | 0.0554 | 0.2043 | 0.0672 |  |
| hsa-miR-1244 | 0.22 | 0.04 | 0.05 |  | 0.0033 | 0.0002 | 0.0002 |  |
| hsa-miR-1255b-5p | 0.15 | 0.57 | 0.32 |  | 0.0023 | 0.0036 | 0.0012 |  |
| hsa-miR-125a-5p | 6.44 | 3.89 | 3.43 |  | 0.0960 | 0.0249 | 0.0132 |  |
| hsa-miR-125b-1-3p | 0.50 | 33.00 | 0.27 |  | 0.0075 | 0.2114 | 0.0011 |  |
| hsa-miR-125b-5p | 81.65 | 105.76 | 110.74 |  | 1.2164 | 0.6774 | 0.4281 |  |
| hsa-miR-1260a | 9.13 | 51.67 | 40.87 |  | 0.1360 | 0.3310 | 0.1580 |  |
| hsa-miR-126-3p | 0.28 | 0.06 | 0.01 |  | 0.0041 | 0.0004 | 0.0001 |  |
| hsa-miR-127-3p | 2.55 | 1.08 | 7.05 |  | 0.0380 | 0.0069 | 0.0272 |  |
| hsa-miR-1275 | 52.32 | 27.98 | 20.62 |  | 0.7795 | 0.1792 | 0.0797 |  |
| hsa-miR-1290 | 5.02 | 16.57 | 7.96 |  | 0.0747 | 0.1061 | 0.0308 |  |
| hsa-miR-1291 | 3.23 | 0.86 | 5.36 |  | 0.0482 | 0.0055 | 0.0207 |  |
| hsa-miR-129-2-3p | 0.17 | 1.26 | 0.19 |  | 0.0025 | 0.0081 | 0.0007 |  |
| hsa-miR-130a-3p | 4.21 | 2.98 | 3.66 |  | 0.0627 | 0.0191 | 0.0141 |  |
| hsa-miR-130b-3p | 0.10 | 0.94 | 0.86 |  | 0.0015 | 0.0060 | 0.0033 |  |
| hsa-miR-132-3p | 84.82 | 64.61 | 26.24 |  | 1.2637 | 0.4138 | 0.1015 |  |
| hsa-miR-133a-3p | 1.14 | 1.61 | 1.66 |  | 0.0170 | 0.0103 | 0.0064 |  |
| hsa-miR-134-5p | 0.30 | 0.11 | 0.56 |  | 0.0044 | 0.0007 | 0.0022 |  |
| hsa-miR-137-3p | 5.33 | 4.00 | 4.24 |  | 0.0794 | 0.0256 | 0.0164 |  |
| hsa-miR-138-5p | 7.49 | 9.30 | 26.89 |  | 0.1115 | 0.0596 | 0.1040 |  |
| hsa-miR-139-5p | 0.29 | 0.56 | 0.19 |  | 0.0042 | 0.0036 | 0.0008 |  |
| hsa-miR-140-3p | 0.50 | 0.07 | 0.04 |  | 0.0075 | 0.0005 | 0.0002 |  |
| hsa-miR-140-5p | 3.83 | 0.62 | 0.76 |  | 0.0570 | 0.0039 | 0.0029 |  |
| hsa-miR-143-3p | 6.80 | 5.86 | 3.48 |  | 0.1014 | 0.0375 | 0.0135 |  |
| hsa-miR-145-3p | 0.04 | 0.01 | 0.02 |  | 0.0006 | 0.0000 | 0.0001 |  |
| hsa-miR-145-5p | 54.85 | 34.26 | 43.62 |  | 0.8171 | 0.2195 | 0.1687 |  |
| hsa-miR-146a-5p | 2.57 | 3.00 | 0.47 |  | 0.0382 | 0.0192 | 0.0018 |  |
| hsa-miR-146b-5p | 2.13 | 2.33 | 3.97 |  | 0.0317 | 0.0149 | 0.0154 |  |
| hsa-miR-148a-3p | 1.50 | 1.95 | 3.09 |  | 0.0223 | 0.0125 | 0.0119 |  |
| hsa-miR-149-5p | 2.15 | 2.04 | 1.24 |  | 0.0320 | 0.0130 | 0.0048 |  |
| hsa-miR-151a-5p | 0.09 | 0.07 | 0.17 |  | 0.0013 | 0.0005 | 0.0007 |  |
| hsa-miR-152-3p | 22.89 | 9.94 | 15.59 |  | 0.3409 | 0.0637 | 0.0603 |  |
| hsa-miR-155-5p | 3.17 | 7.48 | 3.39 |  | 0.0472 | 0.0479 | 0.0131 |  |
| hsa-miR-15b-5p | 4.69 | 1.94 | 3.93 |  | 0.0699 | 0.0124 | 0.0152 |  |
| hsa-miR-16-5p | 12.59 | 14.28 | 7.98 |  | 0.1876 | 0.0914 | 0.0308 |  |
| hsa-miR-17-5p | 53.02 | 70.26 | 38.99 |  | 0.7898 | 0.4500 | 0.1507 |  |
| hsa-miR-181a-2-3p | 1.77 | 0.21 | 0.47 |  | 0.0263 | 0.0013 | 0.0018 |  |
| hsa-miR-181a-3p | 0.02 | 0.30 | 0.02 |  | 0.0003 | 0.0020 | 0.0001 |  |
| hsa-miR-181a-5p | 8.37 | 4.05 | 6.54 |  | 0.1247 | 0.0260 | 0.0253 |  |
| hsa-miR-181c-5p | 0.00 | 0.01 | 0.04 |  | 0.0001 | 0.0001 | 0.0001 |  |
| hsa-miR-186-5p | 3.86 | 4.49 | 2.33 |  | 0.0575 | 0.0288 | 0.0090 |  |
| hsa-miR-191-5p | 91.29 | 201.50 | 58.04 |  | 1.3600 | 1.2906 | 0.2244 |  |
| hsa-miR-192-5p | 0.89 | 2.49 | 1.01 |  | 0.0133 | 0.0160 | 0.0039 |  |
| hsa-miR-193a-5p | 5.28 | 3.81 | 8.94 |  | 0.0787 | 0.0244 | 0.0346 |  |
| hsa-miR-193b-3p | 560.04 | 202.20 | 682.63 |  | 8.3432 | 1.2951 | 2.6392 |  |
| hsa-miR-195-5p | 1.30 | 1.83 | 0.44 |  | 0.0194 | 0.0118 | 0.0017 |  |
| hsa-miR-197-3p | 37.08 | 91.62 | 54.23 |  | 0.5523 | 0.5869 | 0.2097 |  |
| hsa-miR-198 | 0.04 | 0.03 | 0.19 |  | 0.0007 | 0.0002 | 0.0007 |  |
| hsa-miR-199a-3p | 9.63 | 3.94 | 16.48 |  | 0.1434 | 0.0252 | 0.0637 |  |
| hsa-miR-19a-3p | 0.81 | 1.21 | 1.08 |  | 0.0121 | 0.0078 | 0.0042 |  |
| hsa-miR-19b-3p | 52.80 | 91.37 | 50.49 |  | 0.7865 | 0.5852 | 0.1952 |  |
| hsa-miR-203a-3p | 0.70 | 0.63 | 0.60 |  | 0.0105 | 0.0040 | 0.0023 |  |
| hsa-miR-204-5p | 0.31 | 0.11 | 0.14 |  | 0.0046 | 0.0007 | 0.0006 |  |
| hsa-miR-20a-5p | 33.28 | 41.20 | 27.59 |  | 0.4957 | 0.2639 | 0.1067 |  |
| hsa-miR-20b-5p | 0.08 | 0.03 | 0.01 |  | 0.0011 | 0.0002 | 0.0001 |  |
| hsa-miR-210-3p | 67.67 | 46.52 | 44.14 |  | 1.0081 | 0.2979 | 0.1707 |  |
| hsa-miR-212-3p | 6.45 | 7.69 | 2.60 |  | 0.0960 | 0.0493 | 0.0101 |  |
| hsa-miR-214-3p | 19.61 | 34.77 | 129.78 |  | 0.2921 | 0.2227 | 0.5018 |  |
| hsa-miR-21-5p | 76.98 | 51.76 | 63.78 |  | 1.1468 | 0.3315 | 0.2466 |  |
| hsa-miR-218-5p | 1.22 | 1.56 | 3.89 |  | 0.0182 | 0.0100 | 0.0150 |  |
| hsa-miR-221-3p | 72.37 | 45.12 | 80.56 |  | 1.0782 | 0.2890 | 0.3115 |  |
| hsa-miR-222-3p | 1015.80 | 218.37 | 324.48 |  | 15.1327 | 1.3987 | 1.2545 |  |
| hsa-miR-223-3p | 0.22 | 0.94 | 0.13 |  | 0.0032 | 0.0060 | 0.0005 |  |
| hsa-miR-224-5p | 3.20 | 0.99 | 0.37 |  | 0.0477 | 0.0063 | 0.0014 |  |
| hsa-miR-25-3p | 0.31 | 0.34 | 0.53 |  | 0.0046 | 0.0022 | 0.0021 |  |
| hsa-miR-26a-5p | 34.21 | 44.93 | 15.26 |  | 0.5097 | 0.2878 | 0.0590 |  |
| hsa-miR-26b-5p | 6.00 | 6.63 | 3.39 |  | 0.0894 | 0.0425 | 0.0131 |  |
| hsa-miR-27a-3p | 2.73 | 0.53 | 1.96 |  | 0.0406 | 0.0034 | 0.0076 |  |
| hsa-miR-27a-5p | 0.38 | 0.05 | 0.31 |  | 0.0056 | 0.0003 | 0.0012 |  |
| hsa-miR-27b-3p | 2.53 | 1.39 | 3.46 |  | 0.0377 | 0.0089 | 0.0134 |  |
| hsa-miR-27b-5p | 0.13 | 0.07 | 0.05 |  | 0.0020 | 0.0004 | 0.0002 |  |
| hsa-miR-28-3p | 12.93 | 3.84 | 6.90 |  | 0.1926 | 0.0246 | 0.0267 |  |
| hsa-miR-28-5p | 3.18 | 1.62 | 1.15 |  | 0.0474 | 0.0103 | 0.0044 |  |
| hsa-miR-296-5p | 1.19 | 1.37 | 2.19 |  | 0.0178 | 0.0088 | 0.0084 |  |
| hsa-miR-29a-3p | 76.34 | 35.23 | 16.61 |  | 1.1373 | 0.2256 | 0.0642 |  |
| hsa-miR-29b-3p | 3.29 | 2.35 | 0.09 |  | 0.0490 | 0.0151 | 0.0003 |  |
| hsa-miR-301a-3p | 1.30 | 0.24 | 0.05 |  | 0.0193 | 0.0015 | 0.0002 |  |
| hsa-miR-30a-3p | 11.84 | 8.90 | 2.35 |  | 0.1764 | 0.0570 | 0.0091 |  |
| hsa-miR-30a-5p | 11.90 | 3.64 | 2.65 |  | 0.1772 | 0.0233 | 0.0102 |  |
| hsa-miR-30b-5p | 20.86 | 40.58 | 31.38 |  | 0.3107 | 0.2599 | 0.1213 |  |
| hsa-miR-30c-5p | 8.64 | 33.49 | 35.48 |  | 0.1287 | 0.2145 | 0.1372 |  |
| hsa-miR-30e-3p | 4.06 | 4.25 | 1.10 |  | 0.0604 | 0.0272 | 0.0043 |  |
| hsa-miR-31-3p | 2.92 | 5.83 | 2.95 |  | 0.0435 | 0.0373 | 0.0114 |  |
| hsa-miR-31-5p | 80.03 | 41.63 | 63.78 |  | 1.1922 | 0.2667 | 0.2466 |  |
| hsa-miR-320a-3p | 168.13 | 121.57 | 142.51 |  | 2.5046 | 0.7787 | 0.5510 |  |
| hsa-miR-324-3p | 0.80 | 0.03 | 0.11 |  | 0.0119 | 0.0002 | 0.0004 |  |
| hsa-miR-328-3p | 8.24 | 9.94 | 26.61 |  | 0.1227 | 0.0637 | 0.1029 |  |
| hsa-miR-335-5p | 20.48 | 15.14 | 12.23 |  | 0.3052 | 0.0970 | 0.0473 |  |
| hsa-miR-339-3p | 0.73 | 0.29 | 0.28 |  | 0.0109 | 0.0018 | 0.0011 |  |
| hsa-miR-339-5p | 4.10 | 4.17 | 14.32 |  | 0.0611 | 0.0267 | 0.0554 |  |
| hsa-miR-340-3p | 0.09 | 0.08 | 0.05 |  | 0.0013 | 0.0005 | 0.0002 |  |
| hsa-miR-342-3p | 12.15 | 8.91 | 6.35 |  | 0.1811 | 0.0571 | 0.0245 |  |
| hsa-miR-345-5p | 0.53 | 0.09 | 0.40 |  | 0.0078 | 0.0006 | 0.0016 |  |
| hsa-miR-34a-3p | 2.69 | 1.54 | 3.52 |  | 0.0401 | 0.0098 | 0.0136 |  |
| hsa-miR-34a-5p | 9.61 | 22.97 | 14.04 |  | 0.1431 | 0.1471 | 0.0543 |  |
| hsa-miR-34b-3p | 1.09 | 0.64 | 0.71 |  | 0.0163 | 0.0041 | 0.0027 |  |
| hsa-miR-361-5p | 0.21 | 0.44 | 0.94 |  | 0.0031 | 0.0028 | 0.0037 |  |
| hsa-miR-365a-3p | 10.49 | 13.00 | 8.08 |  | 0.1562 | 0.0833 | 0.0312 |  |
| hsa-miR-370-3p | 6.27 | 3.91 | 6.64 |  | 0.0933 | 0.0250 | 0.0257 |  |
| hsa-miR-374a-5p | 4.73 | 3.13 | 2.73 |  | 0.0704 | 0.0200 | 0.0106 |  |
| hsa-miR-376a-3p | 2.94 | 2.22 | 3.24 |  | 0.0438 | 0.0142 | 0.0125 |  |
| hsa-miR-376c-3p | 3.22 | 3.85 | 2.93 |  | 0.0480 | 0.0247 | 0.0113 |  |
| hsa-miR-409-3p | 15.73 | 17.99 | 58.77 |  | 0.2343 | 0.1153 | 0.2272 |  |
| hsa-miR-410-3p | 0.38 | 0.74 | 0.97 |  | 0.0057 | 0.0047 | 0.0037 |  |
| hsa-miR-411-5p | 1.40 | 0.22 | 1.35 |  | 0.0209 | 0.0014 | 0.0052 |  |
| hsa-miR-423-5p | 0.89 | 3.57 | 2.05 |  | 0.0132 | 0.0229 | 0.0079 |  |
| hsa-miR-425-3p | 0.16 | 0.13 | 0.09 |  | 0.0024 | 0.0008 | 0.0004 |  |
| hsa-miR-432-3p | 0.16 | 0.02 | 0.36 |  | 0.0025 | 0.0001 | 0.0014 |  |
| hsa-miR-452-5p | 0.24 | 0.28 | 0.06 |  | 0.0036 | 0.0018 | 0.0002 |  |
| hsa-miR-454-3p | 0.49 | 0.41 | 3.96 |  | 0.0073 | 0.0027 | 0.0153 |  |
| hsa-miR-455-5p | 0.41 | 0.92 | 0.81 |  | 0.0061 | 0.0059 | 0.0031 |  |
| hsa-miR-483-5p | 121.46 | 27.13 | 7.87 |  | 1.8095 | 0.1738 | 0.0304 |  |
| hsa-miR-487b-3p | 0.18 | 0.11 | 0.25 |  | 0.0027 | 0.0007 | 0.0010 |  |
| hsa-miR-491-5p | 0.38 | 0.31 | 0.55 |  | 0.0056 | 0.0020 | 0.0021 |  |
| hsa-miR-493-3p | 0.00 | 0.04 | 0.10 |  | 0.0001 | 0.0002 | 0.0004 |  |
| hsa-miR-494-3p | 4.61 | 1.19 | 3.51 |  | 0.0686 | 0.0076 | 0.0136 |  |
| hsa-miR-495-3p | 0.89 | 1.06 | 0.49 |  | 0.0132 | 0.0068 | 0.0019 |  |
| hsa-miR-520b-3p | 0.00 | 0.13 | 0.01 |  | 0.0000 | 0.0008 | 0.0001 |  |
| hsa-miR-520c-3p | 98.59 | 175.39 | 1.57 |  | 1.4688 | 1.1234 | 0.0061 |  |
| hsa-miR-520e-3p | 10.65 | 2729.92 | 10982.88 |  | 0.1586 | 17.4856 | 42.4626 |  |
| hsa-miR-532-3p | 0.80 | 0.24 | 1.16 |  | 0.0119 | 0.0015 | 0.0045 |  |
| hsa-miR-532-5p | 2.73 | 0.96 | 3.59 |  | 0.0407 | 0.0061 | 0.0139 |  |
| hsa-miR-539-5p | 0.03 | 0.17 | 0.17 |  | 0.0005 | 0.0011 | 0.0006 |  |
| hsa-miR-551b-3p | 22.81 | 31.03 | 613.95 |  | 0.3398 | 0.1988 | 2.3737 |  |
| hsa-miR-572 | 0.34 | 1.28 | 0.63 |  | 0.0051 | 0.0082 | 0.0024 |  |
| hsa-miR-574-3p | 75.87 | 126.29 | 56.06 |  | 1.1302 | 0.8089 | 0.2168 |  |
| hsa-miR-590-5p | 0.21 | 0.52 | 0.23 |  | 0.0032 | 0.0033 | 0.0009 |  |
| hsa-miR-597-5p | 0.04 | 0.15 | 0.14 |  | 0.0006 | 0.0009 | 0.0005 |  |
| hsa-miR-601 | 0.26 | 4818.58 | 0.30 |  | 0.0039 | 30.8639 | 0.0012 |  |
| hsa-miR-625-3p | 0.12 | 0.66 | 0.64 |  | 0.0018 | 0.0042 | 0.0025 |  |
| hsa-miR-628-3p | 0.02 | 0.01 | 0.01 |  | 0.0003 | 0.0001 | 0.0000 |  |
| hsa-miR-629-3p | 0.55 | 0.03 | 0.08 |  | 0.0082 | 0.0002 | 0.0003 |  |
| hsa-miR-636 | 1613.96 | 4759.66 | 10528.19 |  | 24.0439 | 30.4865 | 40.7046 |  |
| hsa-miR-638 | 1.44 | 2.58 | 1.53 |  | 0.0214 | 0.0165 | 0.0059 |  |
| hsa-miR-650 | 1.05 | 1.64 | 1.83 |  | 0.0156 | 0.0105 | 0.0071 |  |
| hsa-miR-660-5p | 3.78 | 3.69 | 2.45 |  | 0.0563 | 0.0237 | 0.0095 |  |
| hsa-miR-663b | 38.14 | 25.80 | 253.87 |  | 0.5683 | 0.1653 | 0.9815 |  |
| hsa-miR-664a-3p | 6.00 | 33.41 | 6.36 |  | 0.0893 | 0.2140 | 0.0246 |  |
| hsa-miR-671-3p | 0.14 | 0.60 | 0.08 |  | 0.0020 | 0.0039 | 0.0003 |  |
| hsa-miR-708-5p | 4.37 | 2.02 | 11.43 |  | 0.0651 | 0.0129 | 0.0442 |  |
| hsa-miR-744-5p | 0.30 | 0.32 | 0.25 |  | 0.0044 | 0.0020 | 0.0010 |  |
| hsa-miR-886-3p | 12.44 | 3.25 | 12.93 |  | 0.1853 | 0.0208 | 0.0500 |  |
| hsa-miR-886-5p | 7.32 | 3.40 | 14.06 |  | 0.1090 | 0.0218 | 0.0544 |  |
| hsa-miR-92a-3p | 31.00 | 31.79 | 29.79 |  | 0.4619 | 0.2036 | 0.1152 |  |
| hsa-miR-93-3p | 0.25 | 0.18 | 0.27 |  | 0.0037 | 0.0012 | 0.0011 |  |
| hsa-miR-93-5p | 1.47 | 0.86 | 1.12 |  | 0.0219 | 0.0055 | 0.0043 |  |
| hsa-miR-939-5p | 2.58 | 13.88 | 16.23 |  | 0.0384 | 0.0889 | 0.0628 |  |
| hsa-miR-942-5p | 0.04 | 0.33 | 0.61 |  | 0.0006 | 0.0021 | 0.0023 |  |
| hsa-miR-99a-5p | 612.43 | 296.66 | 280.91 |  | 9.1236 | 1.9001 | 1.0861 |  |
| hsa-miR-99b-3p | 0.77 | 0.37 | 0.33 |  | 0.0115 | 0.0024 | 0.0013 |  |
| hsa-miR-99b-5p | 22.81 | 7.57 | 19.40 |  | 0.3398 | 0.0485 | 0.0750 |  |

F stands for FBS, P for hPL and S/X for serum/xeno-free medium.
