# Supplementary figures and images for: Secretome and extracellular vesicle signatures in bone marrow-derived mesenchymal stromal cells after expansion in standard and next-generation media
Source: Extracell Vesicles Circ Nucl Acids. 2025 Apr 29;6(2):195–215. doi: 10.20517/evcna.2024.99 (PMC12367461; doi:10.20517/evcna.2024.99)

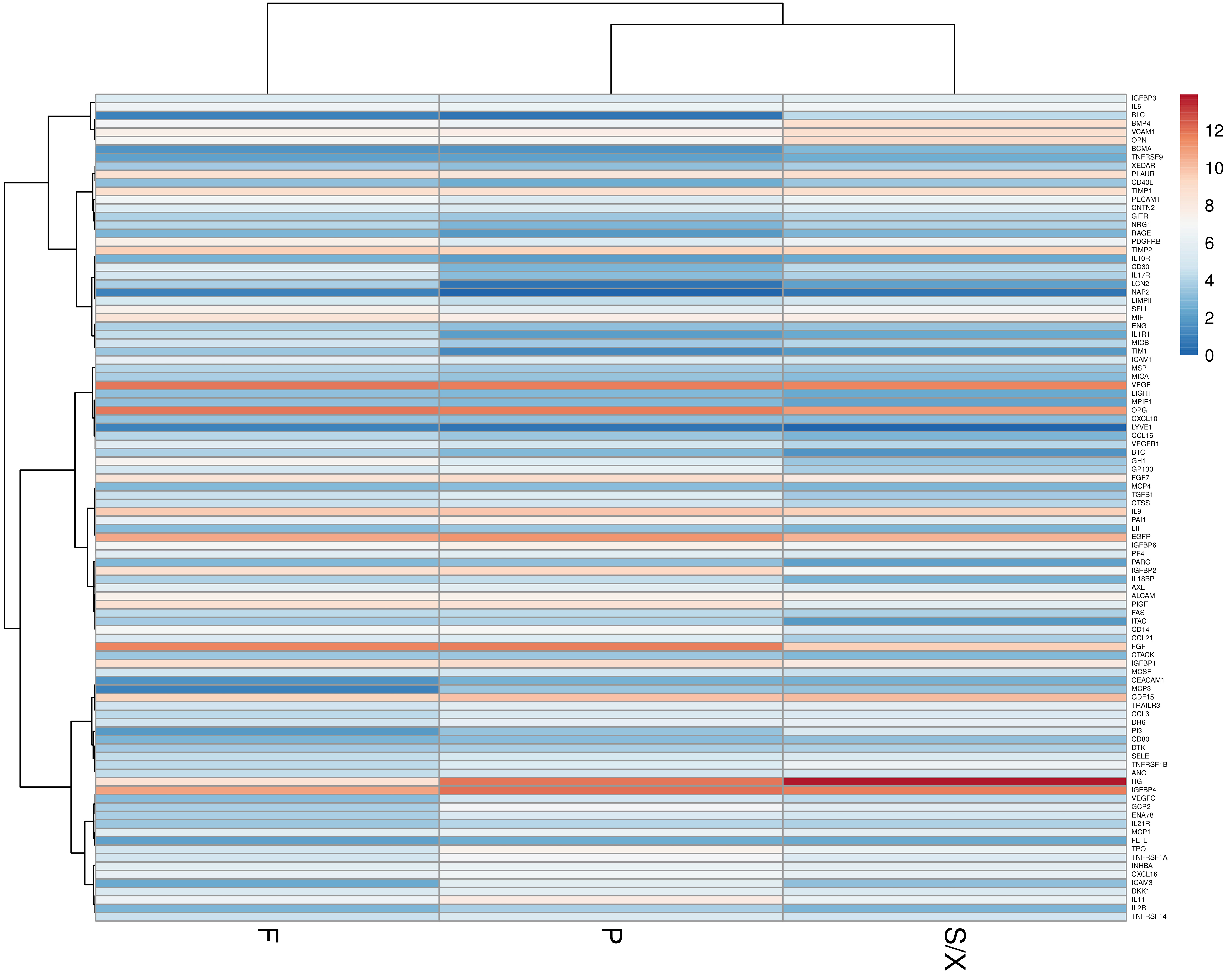

Supplement: Supplementary file 1 [file evcna-6-2-195-SupplementaryMaterials.zip › evcna5099-SupplementaryMaterials/Supplementary Figure 1_EVCNA.jpg]

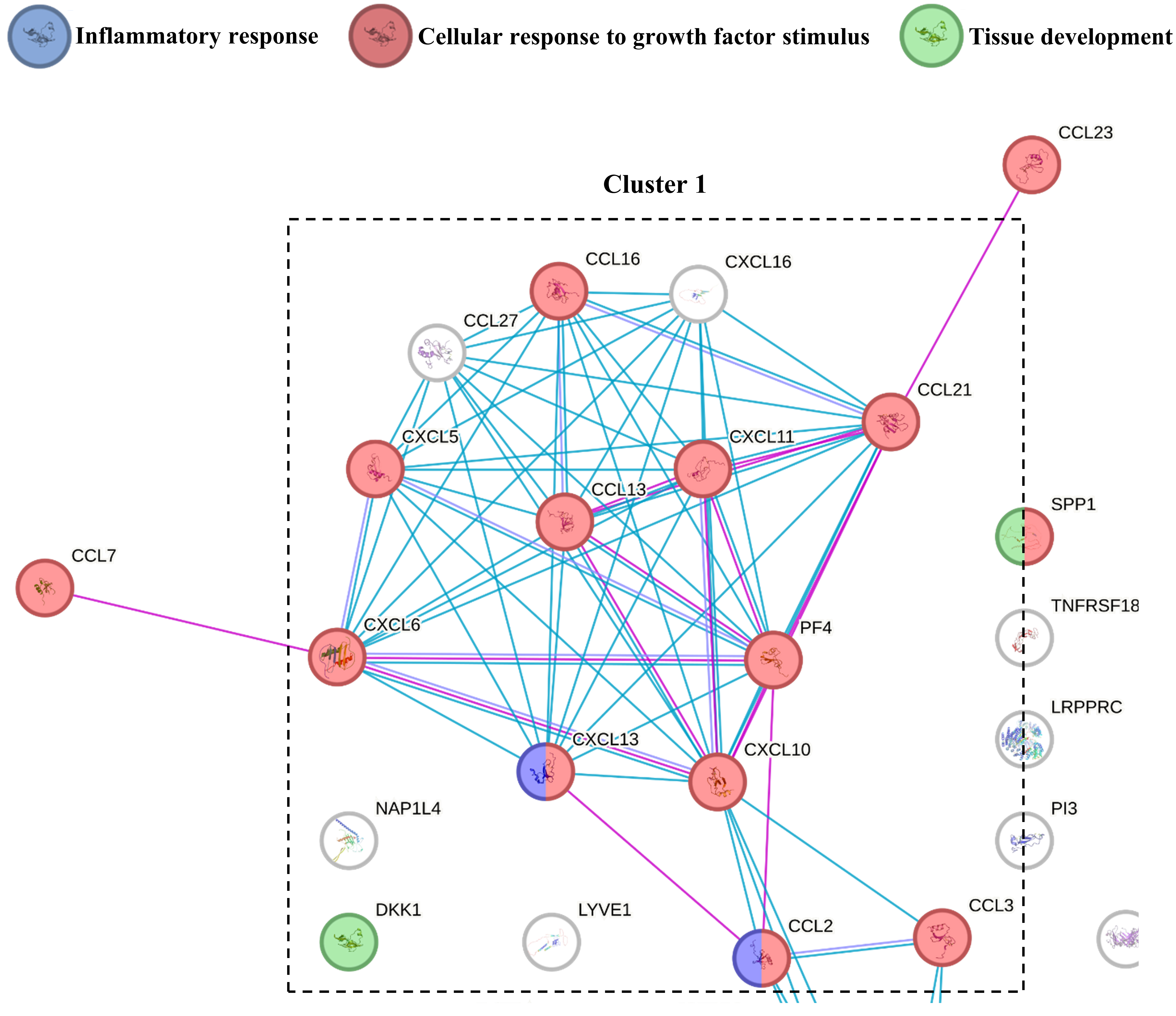

Supplement: Supplementary file 1 [file evcna-6-2-195-SupplementaryMaterials.zip › evcna5099-SupplementaryMaterials/Supplementary Figure 2_EVCNA.jpg]

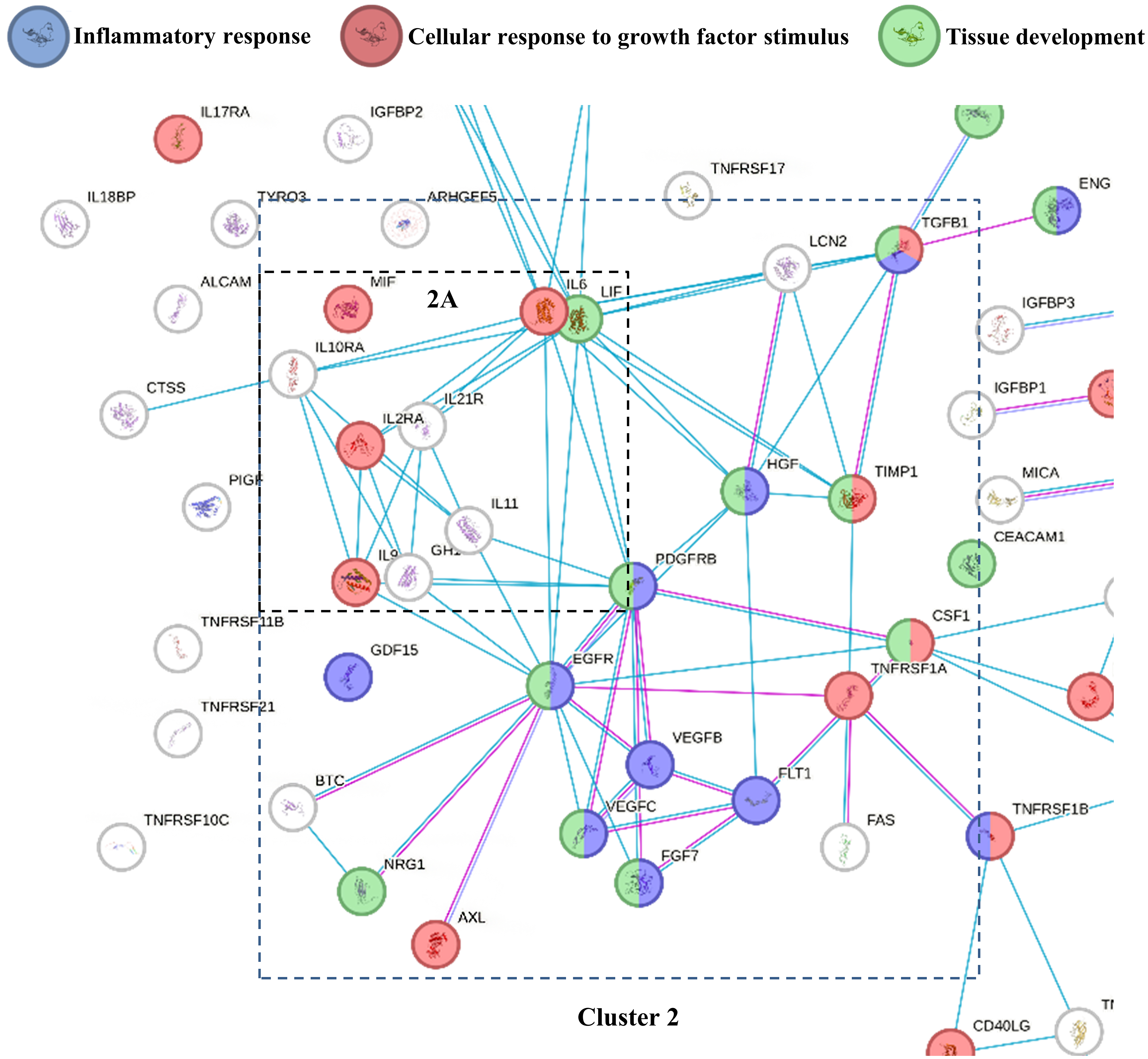

Supplement: Supplementary file 1 [file evcna-6-2-195-SupplementaryMaterials.zip › evcna5099-SupplementaryMaterials/Supplementary Figure 3_EVCNA.jpg]

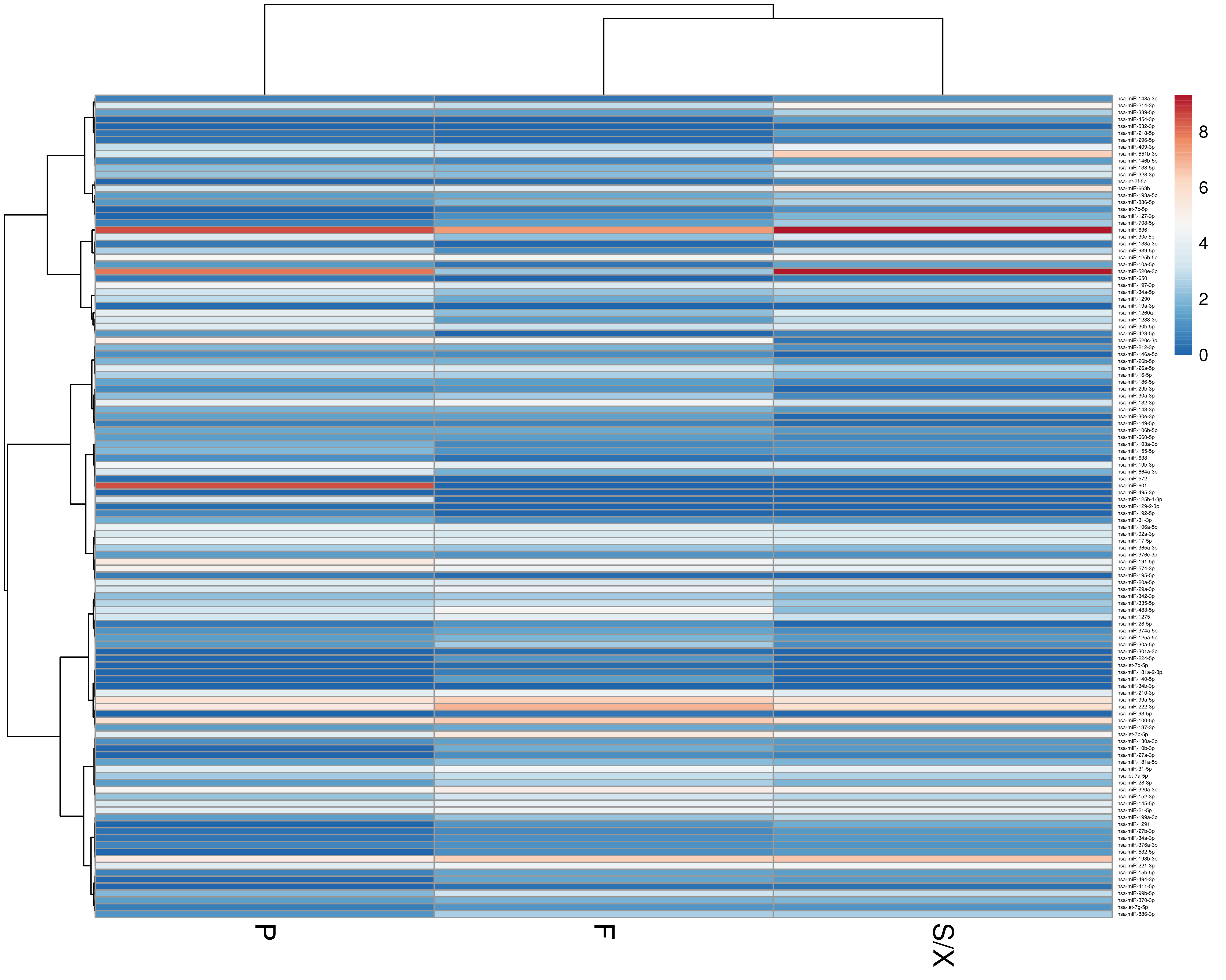

Supplement: Supplementary file 1 [file evcna-6-2-195-SupplementaryMaterials.zip › evcna5099-SupplementaryMaterials/Supplementary Figure 4_EVCNA.jpg]
